# Kombucha-Mediated Fermentation Enhances Antioxidant, Anti-Inflammatory, Anti-Ageing and Antimicrobial Properties of Fruit Tree Leaf Agro-Waste Extracts from *Malus domestica*, *Prunus armeniaca* and *Prunus cerasus*

**DOI:** 10.3390/ijms27125328

**Published:** 2026-06-12

**Authors:** Martyna Zagórska-Dziok, Aleksandra Ziemlewska, Zofia Nizioł-Łukaszewska, Agnieszka Mokrzyńska, Magdalena Wójciak, Justyna Zagórska, Ireneusz Sowa

**Affiliations:** 1 Department of Technology of Cosmetic and Pharmaceutical Products, Medical College, University of Information Technology and Management in Rzeszow, Sucharskiego 2, 35-225 Rzeszow, Poland; aziemlewska@wsiz.edu.pl (A.Z.); zniziol@wsiz.edu.pl (Z.N.-Ł.); amokrzynska@wsiz.edu.pl (A.M.); 2 Department of Analytical Chemistry, Medical University of Lublin, Aleje Raclawickie 1, 20-059 Lublin, Poland; magdalena.wojciak@umlub.pl (M.W.); ireneusz.sowa@umlub.pl (I.S.); 3 Department of Food and Nutrition, Medical University of Lublin, 4a Chodzki Str., 20-093 Lublin, Poland; justyna.zagorska@umlub.edu.pl

**Keywords:** *Malus domestica*, *Prunus armeniaca*, *Prunus cerasus*, kombucha fermentation, agro-waste valorisation, antioxidant activity, antimicrobial activity, anti-inflammatory activity, anti-ageing activity, skin cells

## Abstract

Fruit tree leaves are an abundant agro-waste material with promising yet underexplored biological potential. This study compared the biological activity of aqueous extracts obtained from apple (*Malus domestica*), apricot (*Prunus armeniaca*), and cherry (*Prunus cerasus*) leaves and their kombucha-fermented counterparts in the context of cosmetic and dermatological applications. Phytochemical composition before and after fermentation was analyzed chromatographically. Antioxidant activity was evaluated using DPPH, ABTS, and FRAP assays, while intracellular reactive oxygen species (ROS) levels in keratinocytes and fibroblasts were assessed using the H_2_DCFDA probe. Cytotoxicity was determined by Alamar Blue and Neutral Red assays. Antimicrobial activity against seven bacterial strains was investigated using minimum inhibitory concentration and disc diffusion methods. Anti-inflammatory activity was evaluated in LPS-stimulated THP-1 cells by measuring TNF-α, IL-1β, and IL-6 levels using ELISA. The influence of the samples on collagenase, elastase, and hyaluronidase activity was also analyzed. Fermentation increased the content of selected phenolic compounds and enhanced antioxidant, antimicrobial, anti-inflammatory, and anti-ageing properties. Ferments more effectively reduced oxidative stress in skin cells and showed no cytotoxicity within the tested concentration range. These findings indicate that kombucha fermentation may support the valorization of fruit tree leaf agro-waste as multifunctional ingredients for skincare formulations.

## 1. Introduction

In the context of growing environmental awareness and the principles of a circular economy, plant by-products are increasingly being explored as potential sources of bioactive compounds for applications in cosmetology and pharmacy [1]. Apple (*Malus domestica*), apricot (*Prunus armeniaca*), and cherry (*Prunus cerasus*) leaves, typically regarded as waste material after fruit harvesting, deserve attention as valuable sources of secondary metabolites with diverse biological activities [2].

Previous studies have demonstrated that apple leaves contain polyphenols and flavonoids, such as phloridzin and quercetin, with antioxidant and anti-inflammatory properties and the ability to inhibit key enzymes (COX-1, COX-2) involved in inflammatory processes [3,4]. Moreover, different apple cultivars show variability in polyphenol content and antioxidant capacity [5]. In addition, extracts from apple leaves have been shown to possess antioxidant, anti-inflammatory and genoprotective properties [6]. Apricot leaves have similarly attracted attention for their antioxidant and antimicrobial activities, which have been linked to rich flavonoid and phenolic profiles [2,7]. Polyphenol-rich apricot leaf extracts, notably enriched in quercetin-3-O-rutinoside and 5-O- and 3-O-caffeoylquinic acids, display marked antioxidant activity and inhibit pancreatic lipase and COX-1, indicating potential anti-obesity and anti-inflammatory effects [8]. Seasonal HPLC–DAD profiling of apricot leaves has identified chlorogenic acid, rutin, catechin derivatives, naringin and caffeic acid as key constituents and has shown that their levels vary across maturation stages, which is an important consideration for maximizing bioactive yield [9]. Cherry leaves, particularly those of *Prunus cerasus*, represent an underexploited yet promising source of bioactive compounds. Analyses of *P. cerasus* leaves have shown pronounced cultivar-dependent variation in phenolic composition and antioxidant capacity, with some genotypes characterized by higher levels of total phenolics, chlorogenic acid and flavonols and by particularly strong antiradical and reducing activity in vitro [10]. Studies on sweet cherry (*Prunus avium*) by-products have further demonstrated that leaves and petioles contain higher levels of dietary fibre, vitamin C, carotenoids and polyphenols than the fruit and exhibit stronger antioxidant activity. In these materials, the main phenolic compounds identified include caffeic acid, chlorogenic acid, p-coumaric acid and myricetin [11]. Although the scientific literature on apricot and cherry leaves is relatively limited, available data indicate that they are a source of phenolic compounds, anthocyanins and tannins with anti-inflammatory, antioxidant and anti-ageing properties [8,12,13,14]. As underutilized by-products, these leaves represent promising raw materials for cosmetic applications.

To further enhance their biological value, fermentation processes are increasingly applied, with kombucha fermentation attracting particular attention. Kombucha is a traditional fermented beverage, while the symbiotic culture of bacteria and yeast (SCOBY) used to initiate its production is obtained directly from previous fermentation batches. This kombucha culture is known to transform plant substrates through microbial biotransformation. The traditional kombucha beverage originates from China and is made from sweetened black or green tea. Fermentation begins when yeast hydrolyzes sucrose into glucose and fructose, producing ethanol, which is then converted by acetic acid bacteria into organic acids [15]. This process not only releases and modifies polyphenols, improving their bioavailability, but also generates new bioactive derivatives such as organic acids (such as glucuronic, lactic and acetic acids), vitamins, and other metabolites with antioxidant and antimicrobial properties [16,17]. The generation of these health-promoting compounds occurs through both the microbial synthesis of new metabolites and the enzymatic degradation of the substrate’s native components, where microbial enzymes (such as β-glucosidases) cleave complex glycosides into more active aglycone forms [18]. Optimizing fermentation conditions, such as selecting specific acetic acid bacteria like *Komagataeibacter xylinus* or *K. hansenii*, can significantly increase glucuronic acid production and enhance antioxidant activity. Consequently, glucuronic acid content is frequently used as a key indicator of the biological activity of kombucha extracts [19]. Other reports confirm that kombucha beverages obtained from various substrates (tea leaves, fruits, or other plant materials) demonstrate increased polyphenol content and higher antioxidant activity compared to the unfermented raw material [20,21].

The anti-inflammatory, antioxidant, and cytoprotective properties of plant materials after kombucha fermentation are of particular relevance in the context of skin health. Keratinocytes and fibroblasts, the key cellular components of the epidermis and dermis, are responsible for barrier function, synthesizing and remodelling collagen and elastin fibres and coordinating tissue repair [22,23]. Oxidative stress and inflammation contribute to their damage, driving skin ageing processes [24]. Therefore, plant-derived compounds and kombucha ferments with antioxidant, anti-inflammatory, cytoprotective, and antimicrobial effects are of high interest in the development of anti-ageing and protective skin formulations. The application of kombucha fermentation to by-products such as apple, apricot, and cherry leaves offers an opportunity to obtain products with enhanced biological activity. This approach not only aligns with the principles of sustainable development but also transforms low-value materials into high-value bioresources. Such ferments represent a promising direction for the development of innovative active ingredients for cosmetic and pharmaceutical use [25].

The aim of this study was to characterize the biological properties of non-fermented and kombucha ferments obtained from apple, apricot and cherry leaves, with a focus on their antioxidant, anti-inflammatory, anti-ageing, cytoprotective (towards keratinocytes and fibroblasts) and antimicrobial activities. The findings add to current knowledge on the utilization of unconventional, waste-derived plant materials and their fermented forms as potential bioactive ingredients for cosmetic and pharmaceutical applications.

## 2. Results

### 2.1. Chromatographic Analysis of Phenolic Compounds

**Figure 1:**
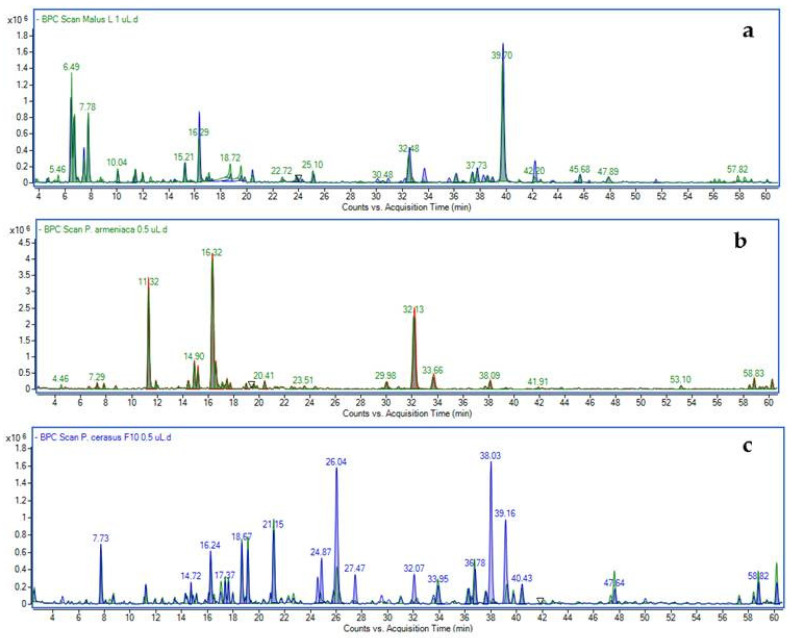
Base peak chromatograms (BPCs) of extracts (green line) from the leaves of (**a**) *Malus domestica*, (**b**) *Prunus armeniaca*, and (**c**) *Prunus cerasus*, overlapped with the corresponding ferments showing the changes after 10 days of fermentation (blue line) and 20 days (red line).

The base peak chromatogram (BPC) profiles obtained from the chromatographic separation of extracts derived from *Prunus armeniaca*, *Prunus cerasus*, and *Malus domestica* are shown in Figure 1. These chromatograms visually represent the most intense ion signals detected during mass spectrometric analysis. Comprehensive mass spectrometry (MS) spectral data are systematically summarized in Tables S1–S3. Preliminary identification of the compounds was based on their mass spectral characteristics and further supported by comparison with previously published data [8,26]. Where available, reference standards were employed to confirm compound identities.

All analyzed extracts are rich in phenolic acids and flavonoids, particularly derivatives of kaempferol and quercetin. In the case of each extract, an increase in metabolites was observed as a result of fermentation. As observed previously, gluconic acid significantly increased in all ferments, because it is a product of sucrose oxidation during fermentation (Figure S1). Moreover, the concentration of gallic acid increased as a result of the hydrolysis of more complex galloylated polyphenols (Figure S2). However, for the remaining compounds, the observed effects depended on the type of extract used as a substrate. In the case of *Prunus cerasus*, a 10-day fermentation period was the most beneficial, and the greatest increases were observed for rutosides of kaempferol, quercetin, and isorhamnetin (approximately 17.4-fold, 7-fold, and 7.3-fold increases, respectively, compared to the non-fermented extract), as well as for derivatives of these aglycones with high molecular weight (*m*/*z*-H = 771 increased by 3.5-fold, *m*/*z*H = 775 by 3.6-fold, and *m*/*z*–H = 785 by 5.9-fold). Phenolic acids remained almost at the same level, although after 20 days of fermentation, a slight decrease in derivatives of *p*-coumaric acids was observed.

In the case of *Malus domestica*, slightly better results were obtained after 20 days of fermentation. However, in general, such significant increases as observed for *P. cerasus* were not noted. The most notable changes were recorded for chlorogenic acid (1.74-fold increase), the aglycone eriodictyol (5-fold increase), and the total amount of *p*-coumaroylquinic acids (1.4-fold increase) compared to non-fermented extract. Similarly, for *P. armeniaca*, the highest increase was observed after 20 days of fermentation. However, the effect of fermentation was the weakest compared to all the analyzed extracts. Only a slight increase was recorded in chlorogenic acid, the total amount of *p*-coumaroylquinic acids, as well as in rutinosides and glucoside. The detailed results of quantification are summarized in Table 1, Table 2 and Table 3. Extracted ion chromatograms within the specific mass ranges for the compounds showing the most pronounced changes are included in the Supplementary Material as Figures S3–S5.

### 2.2. Assessment of Antioxidant Activity

The antioxidant potential of the extracts and fermented samples was evaluated using complementary in vitro assays based on different mechanisms of antioxidant action, including DPPH and ABTS radical scavenging assays as well as the ferric reducing antioxidant power (FRAP) assay. In addition, intracellular reactive oxygen species (ROS) levels were determined in fibroblasts and keratinocytes using the H_2_DCFDA fluorescent probe.

**Figure 2:**
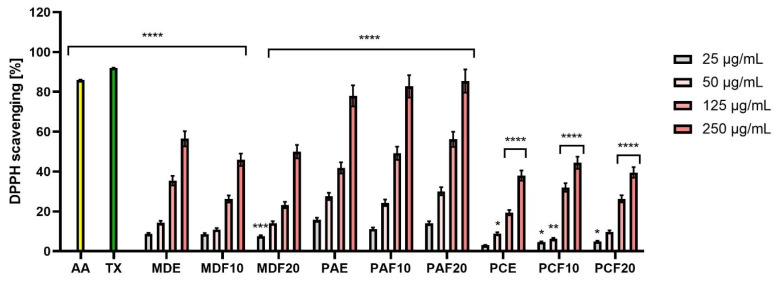
The ability of *M. domestica*, *P. armeniaca* and *P. cerasus* aqueous extracts and ferments (F10 and F20) to scavenge DPPH free radicals at concentrations of 25, 50, 125 and 250 µg/mL. Ascorbic acid (AA; yellow bar; 100 µg/mL) and Trolox (TX; green bar; 100 µg/mL) were used as reference compound. Data are presented as mean ± SD from three independent experiments, with each sample tested in triplicate. **** *p* < 0.0001, *** *p* = 0.0003, ** *p* = 0.0035, * *p* < 0.05.

In the DPPH assay, all investigated samples exhibited concentration-dependent radical scavenging activity (Figure 2). Radical scavenging activity increased with increasing sample concentration, with the highest values generally observed at 250 µg/mL. Fermentation influenced the antioxidant potential of several extracts, although the magnitude of the effect depended on plant species and fermentation time. In the case of *Prunus cerasus*, both fermented samples showed higher activity than the non-fermented extract, with the strongest effect observed after 10 days of fermentation. For *Malus domestica*, radical scavenging capacity gradually increased with fermentation time, particularly at higher concentrations. Among all analyzed samples, the highest DPPH scavenging activity was observed for the 20-day fermented *Prunus armeniaca* extract (PAF20). Comparison of the investigated plant materials showed that antioxidant activity increased in the following order: *P. cerasus* < *M. domestica* < *P. armeniaca*.

**Figure 3:**
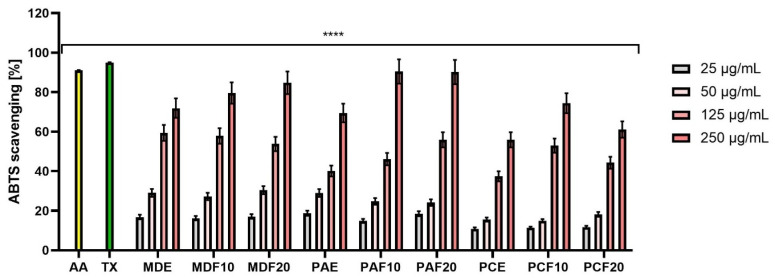
The ability of *M. domestica*, *P. armeniaca*, and *P. cerasus* extracts and ferments (F10 and F20) to scavenge ABTS free radicals at concentrations of 25, 50, 125 and 250 µg/mL. Ascorbic acid (AA; yellow bar;100 µg/mL) and Trolox (TX; green bar; 100 µg/mL) were used as reference compounds. Data are presented as mean ± SD from three independent experiments, with each sample tested in triplicate. **** *p* < 0.0001.

Similar trends were observed in the ABTS assay (Figure 3). All samples demonstrated concentration-dependent antioxidant activity, and fermentation influenced radical scavenging capacity, although the magnitude of the effect depended on plant species and concentration. The positive effect of fermentation was particularly evident for *P. armeniaca* and *P. cerasus* samples at higher concentrations. As in the DPPH assay, *P. armeniaca* samples exhibited the strongest antioxidant activity, whereas *P. cerasus* extracts showed the lowest values.

**Figure 4:**
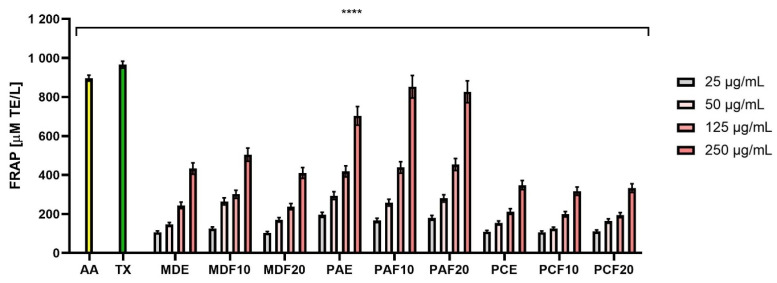
The ability of *M. domestica*, *P. armeniaca* and *P. cerasus* extracts and ferments (F10 and F20) at concentrations of 25, 50, 125 and 250 µg/mL to reduce iron ions, determined by the FRAP method. Ascorbic acid (AA; 100 µg/mL) and Trolox (TX; 100 µg/mL) were used as reference compounds. Data are presented as mean ± SD from three independent experiments, with each sample tested in triplicate. **** *p* < 0.0001.

The FRAP assay confirmed the results obtained using DPPH and ABTS methods (Figure 4). Reducing power increased with increasing concentration for all tested samples. Fermentation influenced ferric reducing ability in selected samples, particularly in fermented *P. armeniaca* extracts. Overall, the antioxidant activity determined by all three chemical assays remained dependent on plant species, fermentation time, and sample concentration. As in the DPPH and ABTS assays, *P. armeniaca* samples generally exhibited the highest reducing power.

Excessive production of reactive oxygen species (ROS) contributes to oxidative stress, leading to damage of membrane lipids, proteins, and nucleic acids, ultimately impairing skin cell function and accelerating skin ageing processes. Therefore, the ability of plant-derived compounds to reduce intracellular ROS levels represents an important indicator of their biological activity in skin-related in vitro models [27,28,29].

**Figure 5:**
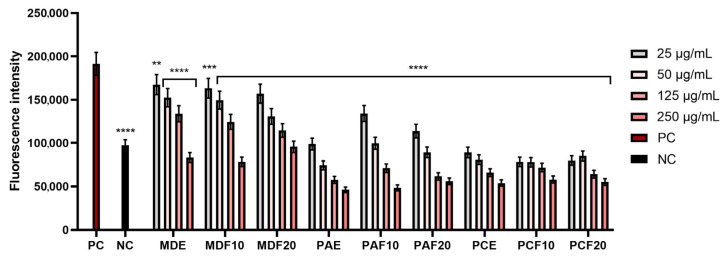
The ability of *M. domestica*, *P. armeniaca* and *P. cerasus* extracts and ferments (F10 and F20) at the concentrations of 25, 50, 125 and 250 µg/mL on the intracellular level of reactive oxygen species in fibroblasts (HDF cells), where NC is the negative control (cells not treated with the test samples and not treated with H_2_O_2_) and PC is the positive control (not treated with the test samples and treated with H_2_O_2_). Data are presented as mean ± SD from three independent experiments, with each sample tested in triplicate. **** *p* < 0.0001, *** *p* = 0.0003, ** *p* = 0.0026.

In HDF cells, all investigated samples reduced intracellular ROS levels compared with the positive control (Figure 5). In most cases, ROS levels decreased in a concentration-dependent manner. The strongest reduction in intracellular ROS levels was observed for the 20-day *P. armeniaca* ferment at 250 µg/mL. Among the investigated plant materials, *P. armeniaca* samples showed the highest antioxidant potential in fibroblasts, whereas *M. domestica* extracts demonstrated comparatively weaker activity.

**Figure 6:**
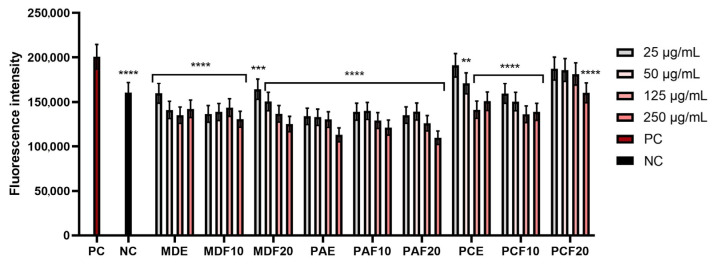
The ability of *M. domestica*, *P. armeniaca* and *P. cerasus* extracts and fermented extracts (F10 and F20) at the concentrations of 25, 50, 125 and 250 µg/mL on the intracellular level of reactive oxygen species in keratinocytes (HaCaT cells), where NC is the negative control (cells not treated with the test samples and not treated with H_2_O_2_) and PC is the positive control (not treated with the test samples and treated with H_2_O_2_). Data are presented as mean ± SD from three independent experiments, with each sample tested in triplicate. **** *p* < 0.0001, *** *p* = 0.0004, ** *p* = 0.0053.

A similar pattern was observed in HaCaT keratinocytes (Figure 6). All tested samples reduced intracellular ROS generation relative to the positive control, although the effect was less pronounced than in fibroblasts. The strongest reduction in intracellular ROS levels was observed for fermented *P. armeniaca* samples, particularly after 20 days of fermentation. In contrast, *P. cerasus* extracts showed the weakest reduction in ROS levels in keratinocytes.

### 2.3. Assessment of Cytotoxicity on Skin Cells In Vitro

Two commonly used tests were employed in the cytotoxicity study of the tested extracts and ferments. In the Alamar Blue test, living, metabolizing cells reduce the non-toxic dye resazurin to highly fluorescent resorufin. The intensity of fluorescence (or colour change) is directly proportional to the metabolic activity of the cell population, providing a rapid and sensitive measurement of viability [30].

The Neutral Red test, on the other hand, is based on the ability of living cells to take up the Neutral Red dye through active intracellular processes and its accumulation in lysosomes. When the integrity of lysosomal membranes is disrupted or other forms of cell damage occur (e.g., due to toxins), dye uptake decreases, which translates into a decrease in the signal read spectrophotometrically [31].

**Figure 7:**
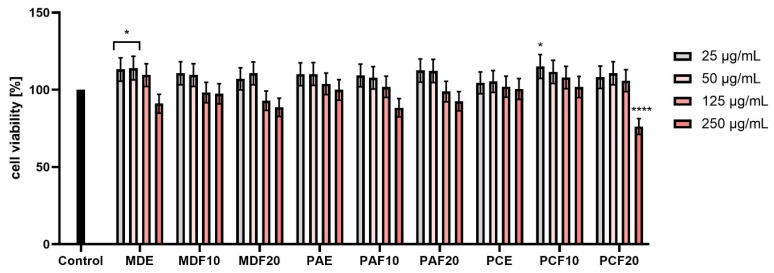
Reduction of resazurin in fibroblasts (HDFs) after 24 h of exposure to the tested extracts and kombucha ferments at concentrations ranging from 25 to 250 µg/mL. The results are presented as the mean ± standard deviation (SD) from three independent experiments, each performed in triplicate. HDF cells not exposed to the tested compounds served as the control, with their viability defined as 100%. **** *p* < 0.0001, * *p* < 0.05.

**Figure 8:**
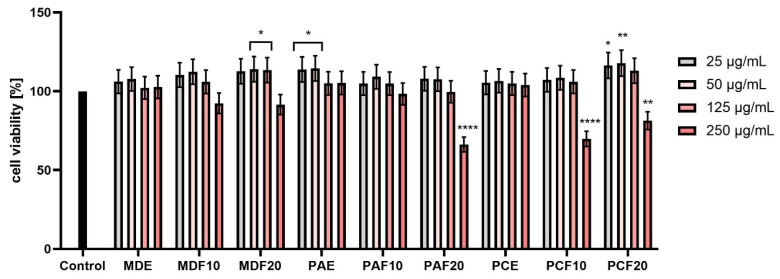
Reduction of resazurin in keratinocytes (HaCaT) after 24 h of exposure to the tested extracts and kombucha ferments at concentrations ranging from 25 to 250 µg/mL. The results are presented as the mean ± standard deviation (SD) from three independent experiments, each performed in triplicate. HaCaT cells not exposed to the tested compounds served as the control, with their viability defined as 100%. **** *p* < 0.0001, ** *p* < 0.01, * *p* < 0.05.

Analysis of the effect of the tested extracts and kombucha ferments on the metabolic activity and viability of skin cells (HDF and HaCaT) showed that in most cases the tested compounds did not cause cytotoxicity, although the effect was concentration-dependent in the range of 25–250 µg/mL. According to the results of the Alamar Blue test (Figure 7 and Figure 8), a gradual decrease in cell viability was observed with increasing concentration, leading to statistically significant cytotoxicity in the case of ferments F10 and F20 at the highest concentration tested. The most favourable values were noted for MDE and PCF10 at a concentration of 25 µg/mL, reaching 113.16% ± 7.59 and 115.13% ± 7.73 of cell viability, respectively, compared to the control (cells not treated with the tested compounds) (Figure 7). Furthermore, after 20 days of fermentation (MDF20 and PCF20) at a concentration of 50 µg/mL, kombucha ferments showed the most favourable effect on cellular metabolic activity, achieving 114.05% ± 7.99 and 117.86% ± 8.26 cell viability, respectively (Figure 8).

**Figure 9:**
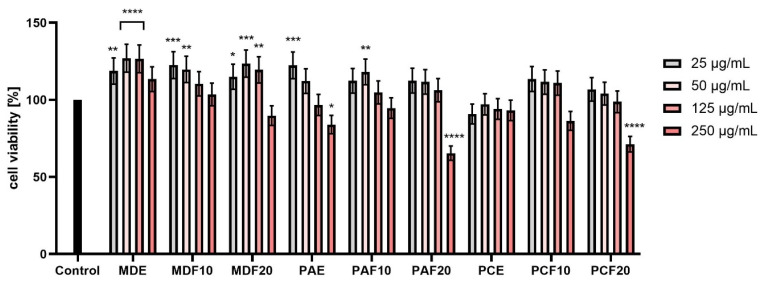
Assessment of Neutral Red uptake in fibroblasts (HDFs) after 24 h of exposure to the tested extracts and kombucha ferments at concentrations ranging from 25 to 250 µg/mL. The results are presented as the mean ± standard deviation (SD) from three independent experiments, each performed in triplicate. HDF cells not exposed to the tested compounds served as the control, with their viability defined as 100%. **** *p* < 0.0001, *** *p* < 0.001, ** *p* < 0.01, * *p* < 0.05.

**Figure 10:**
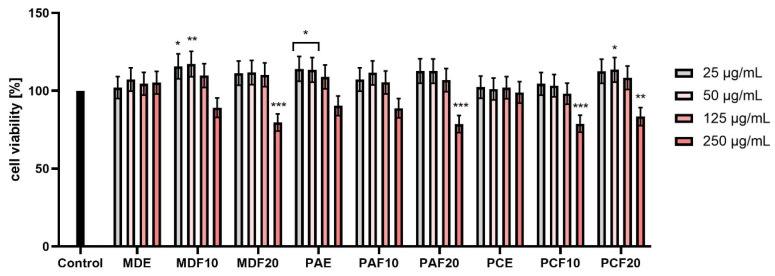
Assessment of Neutral Red uptake in keratinocytes (HaCaT) after 24 h of exposure to the tested extracts and kombucha ferments at concentrations ranging from 25 to 250 µg/mL. The results are presented as the mean ± standard deviation (SD) from three independent experiments, each performed in triplicate. HaCaT cells not exposed to the tested compounds served as the control, with their viability defined as 100%. *** *p* < 0.001, ** *p* < 0.01, * *p* < 0.05.

In the case of the Neutral Red test (Figure 9 and Figure 10), a clear concentration-dependent relationship was observed. Lower doses (25 and 50 µg/mL) promoted cell viability, often reaching statistically significant differences. Both the extract and ferments from *M. domestica* had a beneficial effect on HDF cell viability, reaching approximately 125% cell viability relative to the control at lower concentrations (Figure 9). In the case of the HaCaT line, the most favourable cell viability values were obtained for the MDF10 ferment, which at concentrations of 25 and 50 µg/mL showed 115.69% ± 8.06 and 117.19% ± 8.17 cell viability, respectively (Figure 10).

At the highest concentration used (250 µg/mL), most of the tested ferments caused statistically significant cytotoxicity after 10 and 20 days of fermentation, reaching approximately 75% of cell viability. A likely mechanism may be the degradation of bioactive molecules and the accumulation of adverse fermentation products resulting from the enzymatic activity of microorganisms present in the kombucha consortium [32].

### 2.4. Assessment of Antimicrobial Activity

Aqueous extracts and 10 and 20-day kombucha ferments obtained from leaves of *Malus domestica*, *Prunus armeniaca* and *Prunus cerasus* were evaluated against selected Gram-positive (*Staphylococcus aureus*, *S. epidermidis*, *S. capitis*, *Bacillus subtilis*, *Micrococcus luteus*) and Gram-negative bacteria (*Yersinia enterocolitica*, *Pseudomonas aeruginosa*). In all tested samples, a concentration-dependent antimicrobial effect was observed. Increasing the concentration from 250 to 500 µg/mL led to enlargement of the inhibition zones or to the appearance of measurable inhibition where no effect had been detected at the lower dose. The inhibition zones ranged approximately from 4 to 19 mm, with the highest values recorded for the 20-day ferments. For the majority of strains, ferments exhibited stronger activity than the corresponding aqueous extracts. In Gram-positive bacteria, particularly *S. aureus*, *S. epidermidis* and *S. capitis*, a transition from aqueous extract to 10-day ferment and further to 20-day ferment was associated with a gradual increase in inhibition zone diameters. In several cases (e.g., *M. luteus* and *Y. enterocolitica*), aqueous extracts did not produce measurable zones of inhibition, whereas the ferments, especially after 20 days, generated distinct inhibition zones, indicating the emergence of antimicrobial activity only after fermentation. Within the Gram-positive panel, the largest zones were observed for *S. capitis* and *M. luteus*, in particular for 20-day ferments prepared from *P. armeniaca* and *P. cerasus* leaves (≥15 mm at 500 µg/mL). *B. subtilis* showed moderate susceptibility, with smaller zones overall, yet also in this case 20-day ferments consistently outperformed the corresponding aqueous extracts. Among Gram-negative bacteria, *Y. enterocolitica* and *P. aeruginosa* were more resistant, but the ferments, most prominently the 20-day preparations, still produced inhibition zones reaching approximately 18 to 19 mm, notably for *P. armeniaca* and *M. domestica* leaves at the higher concentration. In several extract and strain combinations no activity was detected for the aqueous extract, while clear inhibition zones appeared after fermentation (Table 4).

The MIC data were consistent with the disc diffusion results. For most strains, MIC values decreased with increasing fermentation time: the highest MICs were obtained for aqueous extracts, lower values for 10-day ferments, and the lowest for 20-day ferments. For staphylococci (*S. aureus*, *S. epidermidis*, *S. capitis*), MICs of the aqueous extracts typically fell within 250 to 400 µg/mL, whereas 20-day ferments showed reduced MICs in the range of 150 to 200 µg/mL, irrespective of the plant species. In some cases (e.g., *S. epidermidis* and *S. capitis* for *P. armeniaca* leaves) the decrease in MIC was particularly pronounced (from 350 to 400 to 150 µg/mL). For *M. luteus*, aqueous extracts were weakly active or inactive (no MIC within the tested range or values up to 600 µg/mL), whereas ferments, especially 20-day preparations, displayed moderate activity (MIC 300 to 500 µg/mL, depending on the plant species). A similar pattern was noted for *Y. enterocolitica*: aqueous extracts from *M. domestica* and *P. cerasus* leaves were inactive at the tested concentrations, while ferments, particularly those from *P. armeniaca* leaves, showed markedly lower MICs (from 400 µg/mL for the extract to 200 µg/mL for the 10-day ferment and 100 µg/mL for the 20-day ferment). *P. aeruginosa* was among the most resistant strains. Although fermentation also reduced MIC values, the MICs of 20-day ferments (200 to 350 µg/mL) remained higher than those determined for the more susceptible Gram-positive bacteria. The lowest MIC for this strain (200 µg/mL) was obtained for 20-day ferments from *M. domestica* and *P. armeniaca* leaves (Table 5).

Taken together, the data demonstrate a clear enhancement of antimicrobial activity after kombucha fermentation, reflected in both increased inhibition zone diameters and decreased MIC values, with the strongest effects generally observed for 20-day ferments and against Gram-positive bacteria.

### 2.5. Anti-Inflammatory Activity

Levels of pro-inflammatory cytokines in THP 1 cells were expressed as fold change relative to the non-stimulated negative control (NC = 1), which consisted of untreated cells cultured under the same experimental conditions without LPS stimulation or exposure to the tested samples. Stimulation with LPS at 10 μg/mL (positive control, PC) induced a marked inflammatory response, with an increase in TNF α of about three-fold, IL 1β of about two-and-a-half-fold and IL 6 of more than fivefold compared with NC. The reference inhibitor diclofenac (D, 10 μg/mL) effectively reduced the LPS-induced production of all three cytokines, bringing TNF-α and IL-1β close to basal values and partially normalizing IL 6, which confirms the responsiveness of the model.

**Figure 11:**
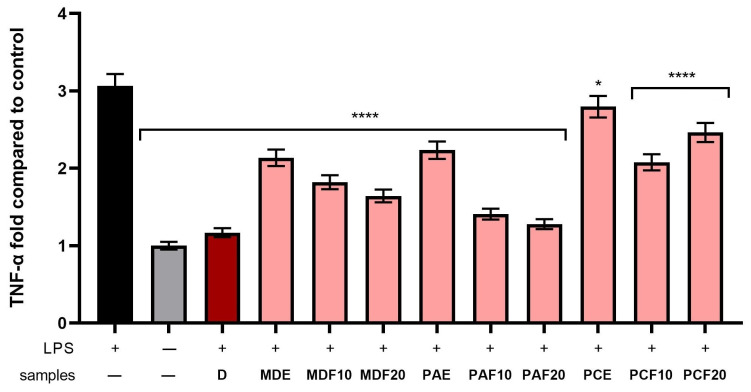
Evaluation of tumour necrosis factor alpha (TNF-α) levels in THP-1 cells after treatment with extracts (at a concentration of 250 µg/mL) from *M. domestica*, *P. armeniaca* and *P. cerasus* before and after 10- (F10) and 20-day (F20) fermentation with kombucha. NC (grey bar) represents the negative control (cells without the tested samples and without LPS), while PC (black bar) represents the positive control (cells not treated with the tested samples but stimulated with LPS). The results are presented as cytokine levels relative to cells not treated with the tested samples (control). Diclofenac (D) at a concentration of 10 µg/mL was used as a reference inhibitor. Bars represent the mean of three independent experiments, in which each sample was tested in duplicate. **** *p* < 0.0001, * *p* < 0.05.

For TNF-α, all aqueous extracts and ferments from apple, apricot and cherry leaves reduced the LPS-driven response compared with PC, although their effects remained weaker than that of diclofenac. Among apple leaf preparations, the aqueous extract (MDWE) maintained TNF-α at around twice the NC value, whereas the 10-day (MDF10) and 20-day (MDF20) ferments further decreased TNF-α to approximately 1.8 and 1.6 times NC, respectively. A similar pattern was observed for apricot leaf samples: the aqueous extract (PAWE) showed values slightly above 2 times NC, while fermented extract, particularly the 20-day preparation (PAF20), lowered TNF-α to about 1.3 times NC, close to the effect of diclofenac. Cherry leaf preparations were generally less effective; the aqueous extract (PCWE) maintained TNF-α at nearly 2.8 times NC and the 10-day fermented extract (PCF10) reduced this to around 2.1 times NC, whereas the 20-day fermented extract (PCF20) showed higher values (about 2.4 times NC) (Figure 11).

**Figure 12:**
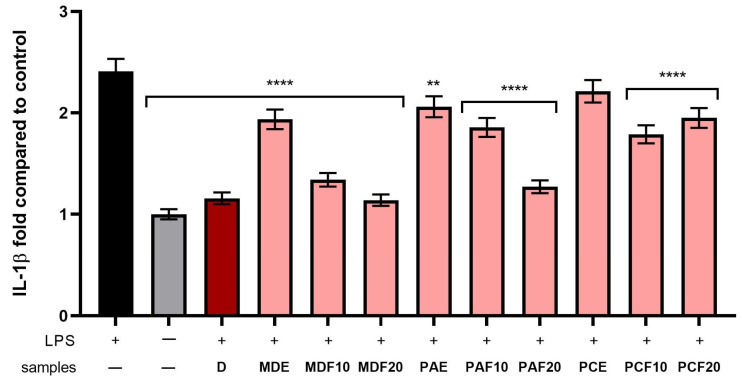
Evaluation of interleukin-1β (IL-1β) levels in THP-1 cells after treatment with extracts (at a concentration of 250 µg/mL) from *M. domestica*, *P. armeniaca* and *P. cerasus* before and after 10- (F10) and 20-day (F20) fermentation with kombucha. NC (grey bar) represents the negative control (cells without the tested samples and without LPS), while PC (black bar) represents the positive control (cells not treated with the tested samples but stimulated with LPS). The results are presented as cytokine levels relative to cells not treated with the tested samples (control). Diclofenac (D) at a concentration of 10 µg/mL was used as a reference inhibitor. Bars represent the mean of three independent experiments, in which each sample was tested in duplicate. **** *p* < 0.0001, ** *p* < 0.01.

The same trend was evident for IL-1β, although the amplitude of changes was smaller. LPS alone increased IL-1β to about 2.4 times NC, and diclofenac reduced this almost to control values. Apple leaf extract kept IL-1β at about 1.9 times NC, and subsequent fermentation gradually decreased cytokine levels, with the 20-day fermented extract reaching values only slightly above NC. Apricot leaf preparations showed a similar pattern and one of the most pronounced effects; the aqueous extract induced IL-1β levels of just above 2 times NC, whereas the 10-day and 20-day ferments gave values of about 1.8 and 1.2 to 1.3 times NC, respectively. In the case of cherry leaves, the aqueous extract and 20-day fermented extract produced IL-1β levels close to 2 times NC, while the 10-day fermented extract lowered IL-1β more effectively (around 1.7 times NC), but still less than the most active apple and apricot ferments (Figure 12).

**Figure 13:**
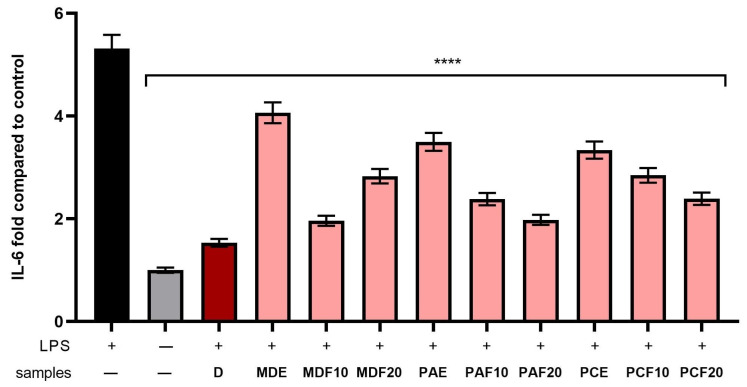
Evaluation of interleukin-6 (IL-6) levels in THP-1 cells after treatment with extracts (at a concentration of 250 µg/mL) from *M. domestica*, *P. armeniaca* and *P. cerasus* before and after 10- (F10) and 20-day (F20) fermentation with kombucha. NC (grey bar) represents the negative control (cells without the tested samples and without LPS), while PC (black bar) represents the positive control (cells not treated with the tested samples but stimulated with LPS). The results are presented as cytokine levels relative to cells not treated with the tested samples (control). Diclofenac (D) at a concentration of 10 µg/mL was used as a reference inhibitor. Bars represent the mean of three independent experiments, in which each sample was tested in duplicate. **** *p* < 0.0001.

For IL-6, LPS caused the strongest induction among the three cytokines (about 5.3 times NC), whereas diclofenac reduced this response to roughly 1.5 times NC. All plant preparations attenuated IL-6 production compared with PC, with clear differences between extracts and ferments. Apple leaf extract (MDWE) maintained IL-6 at around 4 times NC, while the 10-day fermented extract reduced it to about 2 times NC and the 20-day ferment to approximately 2.8 times NC. For apricot leaves, the aqueous extract led to an IL-6 level of about 3.5 times NC, and both ferments produced lower values, with the 20-day ferment giving one of the lowest IL-6 levels in the series (around 2 times NC). Cherry leaf preparations showed intermediate effects: the aqueous extract resulted in an IL-6 level of about 3.3 times NC, while the 10-day and 20-day fermented extract reduced this to roughly 2.8 and 2.4 times NC, respectively (Figure 13).

Overall, the data indicate that all leaf preparations exerted a measurable anti-inflammatory effect in LPS-stimulated THP-1 cells, manifested as a reduction of TNF-α, IL-1β and IL-6 compared with the LPS control. Ferments, in particular the 20-day apricot and apple preparations, generally produced a stronger suppression of cytokine production than the corresponding aqueous extracts, although their activity did not match that of the reference anti-inflammatory drug. Overall, the anti-inflammatory activity depended on both the plant species and fermentation duration, with the strongest effects generally observed for 20-day apricot and apple ferments.

### 2.6. Assessment of Anti-Ageing Activity

**Figure 14:**
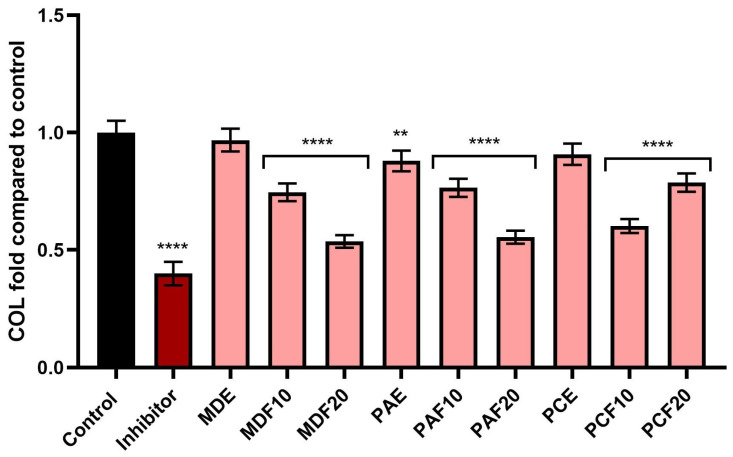
Evaluation of collagenase levels in HDF cells after treatment with extracts (250 µg/mL) from *M. domestica*, *P. armeniaca* and *P. cerasus* before and after 10-day (F10) and 20-day (F20) fermentation with kombucha. Results are presented as enzyme levels relative to cells not treated with the tested samples (control). 1,10-Phenanthroline was used as a reference inhibitor. Bars represent the mean values from three independent experiments, in which each sample was tested in duplicate. **** *p* < 0.0001, ** *p* = 0.0047.

Levels of collagenase, elastase and hyaluronidase in HDF cells were expressed relative to the untreated control, defined as 1.0. All preparations were tested at a concentration of 250 µg/mL. All extracts and ferments from apple, apricot and cherry leaves reduced collagenase levels compared with the control values. For apple leaves, collagenase levels decreased from approximately 0.95 for the aqueous extract to 0.75 and 0.53 for the 10-day and 20-day ferments, respectively. A similar trend was observed for apricot leaf preparations, with values declining from approximately 0.88 for the extract to 0.76 and 0.55 for the corresponding ferments. Cherry leaf samples also reduced collagenase levels, although to a lesser extent. The aqueous extract, 10-day ferment and 20-day ferment yielded values of approximately 0.91, 0.60 and 0.78, respectively. The greatest inhibition of collagenase was observed for the 20-day ferments obtained from apple and apricot leaves. The lowest collagenase levels were observed for the 20-day ferments prepared from apple and apricot leaves, which reduced enzyme levels to approximately half of the control value (Figure 14).

**Figure 15:**
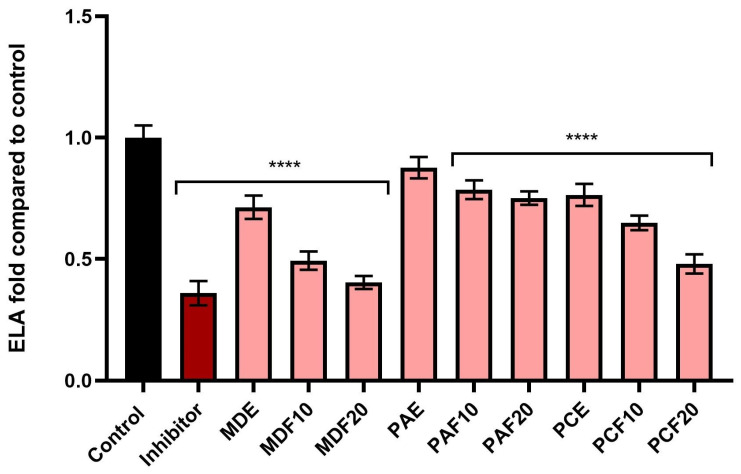
Evaluation of elastase levels in HDF cells after treatment with extracts (250 µg/mL) from *M. domestica*, *P. armeniaca* and *P. cerasus* before and after 10-day (F10) and 20-day (F20) fermentation with kombucha. Results are presented as enzyme levels relative to cells not treated with the tested samples (control). Succinyl-alanyl-alanyl-prolyl-valine-chloromethyl ketone was used as a reference inhibitor. Bars represent the mean values from three independent experiments, in which each sample was tested in duplicate. **** *p* < 0.0001.

A similar pattern was observed for elastase (Figure 15). Apple leaf preparations reduced elastase levels from approximately 0.71 for the extract to 0.50 and 0.40 for the 10-day and 20-day ferments, respectively. Apricot leaf samples exerted a weaker effect, with elastase levels of approximately 0.88, 0.78 and 0.74 for the extract, 10-day ferment and 20-day ferment, respectively. Cherry leaf preparations showed intermediate activity, yielding values of approximately 0.76, 0.66 and 0.49. Among all tested samples, the lowest elastase levels were recorded for the 20-day ferments from apple and cherry leaves, which reduced elastase levels to approximately 40–50% of the control value (Figure 15).

**Figure 16:**
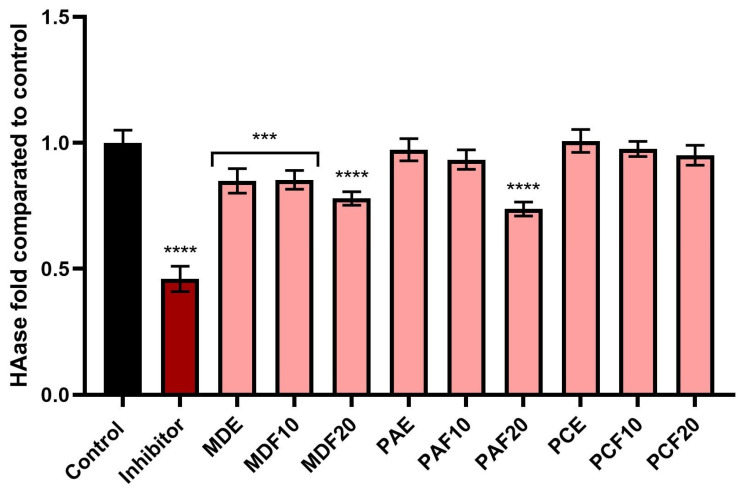
Evaluation of hyaluronidase levels in HDF cells after treatment with extracts (250 µg/mL) from *M. domestica*, *P. armeniaca* and *P. cerasus* before and after 10-day (F10) and 20-day (F20) fermentation with kombucha. Results are presented as enzyme levels relative to cells not treated with the tested samples (control). Tannic acid was used as a reference inhibitor. Bars represent the mean values from three independent experiments, in which each sample was tested in duplicate. **** *p* < 0.0001, *** *p* < 0.001.

Changes in hyaluronidase levels were less pronounced than those observed for collagenase and elastase (Figure 16). Apple leaf preparations produced values of approximately 0.85, 0.87 and 0.78 for the extract, 10-day ferment and 20-day ferment, respectively. Apricot leaf samples showed a greater reduction, with hyaluronidase levels decreasing from approximately 0.97 for the extract to 0.93 and 0.73 for the 10-day and 20-day ferments, respectively. In contrast, cherry leaf preparations had only a limited effect, with values remaining close to the control level (approximately 1.0, 0.97 and 0.95 for the extract, 10-day ferment and 20-day ferment, respectively). The lowest hyaluronidase levels were observed for the 20-day apricot ferment (Figure 16).

Overall, all leaf-derived preparations reduced collagenase and elastase levels in HDF cells, with fermentation generally strengthening this effect, particularly after 20 days. The influence on hyaluronidase levels was less pronounced and depended more strongly on the plant material. The greatest reduction in hyaluronidase levels was observed for apricot leaf ferments, whereas apple leaf preparations showed a moderate effect and cherry leaf preparations produced only minor changes.

Taken together, these results show that all leaf preparations at 250 µg/mL exerted a noticeable inhibitory effect on collagenase and elastase in HDF cells, with fermentation, particularly for 20 days, generally enhancing this activity. The impact on hyaluronidase was weaker and more preparation-dependent, with the most relevant inhibition observed for the 20-day ferments from apricot and, to a lesser extent, apple leaves.

## 3. Discussion

This study demonstrates that aqueous extracts and kombucha ferments obtained from leaves of *Malus domestica*, *Prunus armeniaca* and *Prunus cerasus* constitute a potential source of multifunctional bioactive ingredients with relevance for topical applications. By combining detailed chromatographic profiling with a broad biological evaluation, including antioxidant, antimicrobial, anti-inflammatory and anti-ageing assays in skin-related cell models, we show that kombucha fermentation can substantially modulate both the phenolic composition and the biological activity of these underutilized waste materials.

The chromatographic data confirmed that all three leaf types are rich in phenolic acids and flavonoids, particularly derivatives of kaempferol and quercetin, in agreement with previous reports on apple, apricot and sour cherry foliage [8,10,33]. Fermentation with a SCOBY culture led to a marked increase in several metabolites, including gluconic acid, which is a typical product of sucrose oxidation during kombucha fermentation, and gallic acid, most likely formed through hydrolysis of galloylated polyphenols [19,34]. In *P. cerasus* leaves, the 10-day fermentation was especially effective in enhancing the levels of kaempferol, quercetin and isorhamnetin rutinosides and high-molecular-mass derivatives, while *M. domestica* and *P. armeniaca* required a longer, 20-day fermentation to reach their highest enrichment in selected compounds such as chlorogenic acid, eriodictyol and *p*-coumaroylquinic acids. These observations illustrate that the impact of kombucha fermentation on phenolic profiles is highly matrix-dependent and that the optimal fermentation time is specific for each plant material and target metabolite group. A similar pattern was observed in the biological assays, where the effects of fermentation varied depending on the plant species, fermentation duration and the endpoint analyzed, indicating that phytochemical changes did not always translate directly into proportional changes in biological activity.

The changes in phenolic composition were reflected in a clear functional response in antioxidant assays. To comprehensively evaluate the antioxidant potential of the investigated extracts and ferments, complementary in vitro assays based on different reaction mechanisms were applied. The DPPH and ABTS assays evaluate free radical scavenging ability, whereas the FRAP method reflects the reducing power of the tested samples. The combined use of these assays enables a broader assessment of antioxidant activity associated with both hydrogen atom transfer and electron transfer mechanisms [35]. In cell-free systems (DPPH, ABTS and FRAP), all preparations showed a strong concentration-dependent increase in activity, with the highest effects at 5%. Across all three methods, the order of potency was generally consistent, with apricot leaf samples exhibiting the highest antioxidant activity, followed by apple leaves, whereas cherry leaf preparations showed the lowest values. Fermentation further enhanced these effects, in all plant materials, although the magnitude of improvement depended on plant species and fermentation time. In particular, *M. domestica* showed a marked increase after 20 days of fermentation, while the overall highest antioxidant activity was still observed for *P. armeniaca* preparations. For *P. cerasus* and *P. armeniaca*, the benefit of fermentation depended on both the assay used and the concentration tested. Although chemical antioxidant assays provide valuable information regarding radical scavenging and reducing properties, cell-based ROS assays better reflect the biological relevance of antioxidant activity under physiological conditions. The intracellular ROS assays in HDF and HaCaT cells confirmed that these antioxidant effects are not limited to chemical scavenging in solution but extend to the mitigation of oxidative stress in skin-relevant cell models. In both cell lines, all preparations reduced stimulus-induced ROS generation below the level of the positive control, with the most pronounced reduction observed for 20-day fermented apricot leaf preparations (PAF20), particularly at the highest concentrations tested. Given the central role of oxidative stress in photoaging and in the disruption of epidermal and dermal homeostasis, the combination of strong chemical antioxidant capacity with ROS-lowering effects observed in keratinocytes and fibroblasts suggests that these ferments may be of interest for further investigation as anti-ageing cosmetic ingredients [24]. The strong antioxidant performance of the investigated preparations is consistent with the high phenolic content reported for fruit tree leaves. Apricot (*Prunus armeniaca*) leaves have been described as a rich source of chlorogenic acid and caffeic acid derivatives, as well as quercetin glycosides with pronounced antioxidant activity [36]. Similar observations have been made for sour cherry (*Prunus cerasus*) leaves, which contain substantial amounts of phenolic acids, primarily chlorogenic acid, and flavonols, resulting in high antioxidant potential [10]. Apple (*Malus domestica*) leaves are particularly abundant in quercetin and its derivatives, present at much higher levels in leaves than in the corresponding fruits [33]. These literature data support the view that the high antioxidant capacity observed for our preparations may be associated with their phenolic profiles. However, because no correlation analysis between individual metabolites and antioxidant activity was performed, the contribution of specific compounds should be interpreted with caution. In addition, several studies have shown that fermentation of plant materials with a symbiotic culture of bacteria and yeast (SCOBY) can increase the bioavailability and biological activity of phenolic compounds [37]. The polyphenols and other bioactive constituents present in apricot, cherry and apple leaves provide these raw materials with a high intrinsic antioxidant potential, which can be further enhanced through kombucha fermentation. The biotransformation process promotes the release and structural modification of phenolics, improves their accessibility and leads to the formation of additional antioxidant metabolites [38,39]. The enhanced activity observed after fermentation may result from enzymatic biotransformation of glycosylated polyphenols into lower-molecular-weight aglycones characterized by improved bioavailability and biological activity. Microbial β-glucosidases produced by yeasts and acetic acid bacteria present in SCOBY cultures are known to hydrolyse flavonoid glycosides, thereby increasing the levels of more biologically active compounds such as quercetin or kaempferol aglycones [16]. Consequently, kombucha fermentation may enhance the antioxidant properties of fruit tree leaves, although the magnitude of this effect depends on the plant species, fermentation time and the antioxidant endpoint evaluated.

Importantly, the bioactivities observed in cellular models were achieved at concentrations that did not induce marked cytotoxicity in either HDF or HaCaT cells, as confirmed by Alamar Blue and Neutral Red assays. While some decrease in viability at the highest concentrations and for the longest fermentation times cannot be excluded, the overall pattern indicates a reasonable safety margin for the doses used in antioxidant, ROS, anti-inflammatory and anti-ageing experiments. This cytocompatibility is essential when considering potential cosmetic applications and suggests that, when appropriately formulated and neutralized to skin-compatible pH, kombucha ferments from fruit tree leaves may warrant further investigation for potential incorporation into topical formulations without adversely affecting epidermal or dermal cell viability under the tested in vitro conditions. As other researchers have shown, extracts from the plants studied, for example from apples (phenol-rich fractions), reduce UV-induced DNA damage and oxidative stress in cultured human fibroblasts and increase cell survival after UV exposure. This effect is related to antioxidant properties and modulation of apoptosis pathways [40]. Furthermore, experimental studies of apple stem cell extracts (e.g., PhytoCellTec™ *Malus domestica*) have reported skin cell protection and delayed skin ageing in animal models with UV-induced damage [41]. Taking a closer look at other extracts studied, metabolic analyses of *Prunus cerasus* revealed a rich antioxidant profile, including flavonoids, phenolic acids, and anthocyanins, which are known for their anti-inflammatory properties and reactive oxygen species (ROS) scavenging properties. Cellular studies using human keratinocytes demonstrated the extracts’ ability to reduce ROS levels induced by various stimulants, indicating their potential to alleviate inflammation and protect against oxidative stress-induced damage [42]. In turn, apricot (*Prunus armeniaca*) extracts have demonstrated significant positive effects on skin cells due to their rich content of phenolic compounds, flavonoids, vitamins, and essential fatty acids. These compounds exhibit potent antioxidant and anti-inflammatory effects, which may help protect skin cells from oxidative stress, support barrier integrity, and reduce inflammation [43,44]. Bioactive substances such as *p*-coumaroylquinic acid, caffeic acid, gallic acid, and other phenolic compounds are particularly associated with skin protective and regenerative effects [45]. The available literature data show that the presence of many bioactive compounds in the analyzed extracts and ferments may contribute to their beneficial effect on skin cells. Among the metabolites identified in the investigated extracts and ferments, several compounds may be particularly relevant to the biological activities observed in this study. Chlorogenic acid and caffeic acid derivatives are widely recognized for their antioxidant and anti-inflammatory properties and have been reported to reduce intracellular ROS production and modulate inflammatory signalling pathways in skin cells [46]. Quercetin and kaempferol derivatives, which were abundant in all three leaf materials and in some cases increased after fermentation, are known to inhibit NF-κB activation, suppress pro-inflammatory cytokine production and protect cells against oxidative stress-induced damage [47]. In apple leaf preparations, phlorizin and phloridzin may additionally contribute to antioxidant activity due to their documented radical scavenging properties [48]. Furthermore, gallic acid and *p*-coumaroylquinic acid derivatives, whose levels increased following fermentation, have been associated with antioxidant, antimicrobial and enzyme-inhibitory activities, including effects on collagenase and elastase [49,50,51]. Therefore, it is plausible that the enhanced biological activity of fermented samples results from the combined contribution of these metabolites together with other fermentation-derived compounds, rather than from changes in a single phenolic constituent. However, since no correlation analysis between individual metabolites and biological activities was performed in the present study, the contribution of specific compounds should be considered putative and requires further investigation. Therefore, the biological effects observed in this study are likely the result of the combined action of multiple metabolites rather than a single dominant compound. *p*-Coumaric acid and its derivatives reduced the survival of melanoma cells (SK-MEL-37) after prior irradiation, suggesting their selective effect on diseased cells [52]. Catechins, on the other hand, like other flavonols described in the context of photoprotection, were associated with increased fibroblast viability and survival following radiation exposure, indicating its ability to reduce oxidative damage and support repair processes. The mechanism of action is likely related to the inhibition of p38 and JNK phosphorylation [53]. Phlorizin and phloridzin, naturally found in apples, are valued for their strong antioxidant properties and function as a natural UV filter in leaves, which may translate into their potential protective effect in the context of skincare [33]. Quercetin and its derivatives are indicated as promising substances protecting keratinocytes from damage induced by UVB radiation [54]. Numerous studies also emphasize that phenolic compounds, including quercetin, can protect DNA in fibroblasts thanks to their anti-inflammatory properties [55].

Fermentation also had a substantial impact on antimicrobial activity. While the aqueous extracts already exhibited measurable activity against several Gram-positive and Gram-negative strains, both the inhibition zones and MIC values consistently indicated that 10- and particularly 20-day ferments were more potent than their non-fermented counterparts. This pattern was especially clear for staphylococci and *M. luteus*, for which a stepwise increase in inhibition zone diameter was observed when moving from extract to 10-day and then to 20-day ferment, accompanied by a parallel decrease in MIC values. In several combinations (e.g., *M. luteus* and *Y. enterocolitica*), aqueous extracts were inactive within the tested range, whereas the ferments, especially after 20 days, produced distinct inhibition zones and moderate MIC values, indicating that antimicrobial activity emerged only after fermentation. As expected, Gram-negative bacteria such as *P. aeruginosa* were more resistant, yet even in this case the MICs of 20-day ferments were reduced compared with the extracts, with the lowest MICs again recorded for apple and apricot leaves. These findings align with growing evidence that kombucha-type fermentations can enhance antimicrobial properties, likely through a combination of increased acidity (gluconic and other organic acids), the release of more lipophilic aglycones from glycosylated flavonoids and the formation of additional low-molecular-weight metabolites with membrane-perturbing effects [21,56,57]. From a practical perspective, the observed activity against skin-relevant Gram-positive bacteria, coupled with moderate effects against Gram-negative species, suggests that these ferments could contribute to the control of cutaneous microbiota and to the protection of compromised skin, while still requiring careful evaluation of selectivity and microbiome impact in vivo.

In the THP-1 model of LPS-induced inflammation, all leaf preparations exerted a measurable anti-inflammatory effect, manifested as a reduction in TNF-α, IL-1β and IL-6 levels relative to the positive control. Although none of the samples matched the potency of diclofenac, fermentation strengthened the anti-inflammatory properties in most cases. The 20-day ferments from apple and apricot leaves were the most effective overall, reducing TNF-α and IL-1β to values close to basal levels and markedly attenuating IL-6, which was the most strongly induced cytokine in this system. Cherry leaf preparations showed more modest and less consistent effects, with 10-day ferments often performing better than 20-day ferments, mirroring the more complex pattern observed in the phenolic profile. The ability of fermented leaf preparations to reduce the levels of key pro-inflammatory mediators is particularly relevant given that oxidative stress and inflammation are closely interlinked in skin biology [58]. Polyphenols such as chlorogenic acid, quercetin derivatives and other flavonoids present in these leaves are known to interfere with NF-κB and MAPK signalling and to modulate cytokine release [59,60,61] and it is conceivable that the improved anti-inflammatory effects after fermentation are linked to both enhanced bioaccessibility of these compounds and to newly generated metabolites [62].

The anti-ageing assays in HDF cells further supported the favourable profile of the kombucha ferments. At a concentration of 250 µg/mL, all preparations inhibited collagenase and elastase activity below control values, with fermentation generally amplifying the effect. The most pronounced inhibition of collagenase was observed for the 20-day ferments from apple and apricot leaves, which reduced enzyme activity to approximately half of the basal level, whereas cherry ferments exerted a weaker but still relevant effect. A similar pattern was observed for elastase, with 20-day apple and cherry ferments producing the strongest suppression, followed by more moderate inhibition by apricot preparations. In contrast, hyaluronidase activity was only moderately affected. The 20-day apricot ferment exhibited the clearest inhibitory effect, decreasing activity to roughly three quarters of control, while apple ferments induced a weaker, and cherry ferments an almost negligible reduction. This selective targeting of collagenase and elastase with only limited interference with hyaluronidase may be advantageous from a cosmetic perspective. Effective anti-ageing strategies aim to reduce excessive degradation of collagen and elastin, while preserving sufficient hyaluronan turnover to maintain normal extracellular matrix remodelling and water homeostasis [50,63]. In this context, the observed profile suggests that the ferments, particularly from apple and apricot leaves, could support dermal matrix integrity without strongly suppressing hyaluronidase-dependent processes.

Taken together, these findings highlight the potential of kombucha fermentation as a strategy for the utilization of fruit tree leaf biomass. By modulating the phenolic profile and generating additional organic acids and small metabolites, fermentation increased the antioxidant, antimicrobial, anti-inflammatory and anti-ageing activities of leaf preparations, with the strongest antioxidant, anti-inflammatory and anti-hyaluronidase effects generally observed for 20-day apricot ferments, whereas 20-day apple ferments were particularly effective in collagenase and elastase inhibition. At the same time, the data underscore that the optimal fermentation time and the magnitude of the effect depend on the plant species and on the biological endpoint considered: whereas 10-day ferments of cherry leaves showed particularly high levels of specific flavonoid glycosides, 20-day ferments were more advantageous for antimicrobial and enzyme-inhibitory activities of apple and apricot leaves, and the response of cherry preparations in anti-inflammatory and anti-ageing assays was more variable. These nuances emphasize the need for tailored bioprocess optimization for each raw material.

The growing interest in fermented plant-derived ingredients has also been reflected in the cosmetic industry, where kombucha-based extracts and ferments are increasingly incorporated into skincare formulations. Such ingredients can already be found in products such as facial serums, creams, toners and masks, particularly those intended for anti-ageing, antioxidant or soothing applications. Their popularity is associated with the presence of bioactive compounds generated or released during fermentation, including polyphenols, organic acids and other low-molecular-weight metabolites [25,64,65].

The results obtained in the present study suggest that ferments prepared from fruit tree leaves may also be considered as potential ingredients for cosmetic formulations. Apricot leaf ferments, which exhibited strong antioxidant and anti-inflammatory activity, may be particularly interesting for products intended for skin exposed to oxidative stress. Apple leaf ferments showed the greatest reduction in collagenase and elastase levels and could therefore be considered for formulations targeting signs of skin ageing. In turn, the antimicrobial activity observed for several preparations may support their further evaluation in products designed for skin prone to microbiological imbalance. However, additional studies using more advanced skin models and formulation-based experiments are needed to verify whether the biological effects observed in vitro can be maintained in finished cosmetic products.

The main limitations of this work are the in vitro nature of the assays and the focus on a single fermentation system. Skin models based on monolayer cultures, while informative, do not fully capture the complexity of the epidermal barrier, dermal–epidermal interactions or the role of the skin microbiome. Further studies in reconstructed human skin equivalents and ultimately in vivo will be necessary to confirm the potential skin-related benefits suggested by the present findings and to determine appropriate use levels in finished formulations. In addition, characterization of the microbial composition of the SCOBY and systematic analysis of fermentation parameters (temperature, sugar concentration, mixed leaf matrices) could provide further insight into the biotransformation pathways that underlie the observed changes in activity. In future work, we therefore aim to address these issues by extending our investigations to more advanced skin models and in vivo settings, in order to further clarify the mechanisms of action and to critically evaluate the suitability of these non-fermented and kombucha-fermented leaf extracts for dermatological and cosmetological applications.

Despite these limitations, the present data provide a coherent and encouraging set of evidence that leaves of *M. domestica*, *P. armeniaca* and *P. cerasus*, currently treated as low-value agricultural waste, can be transformed via kombucha fermentation into cytocompatible preparations with a broad spectrum of desirable properties for dermocosmetic applications. Their combined antioxidant, antimicrobial, anti-inflammatory and enzyme-inhibitory activities, together with the possibility of sourcing them from existing orchard residues, position these ferments as attractive candidates for further investigation of sustainable, bio-based ingredients for skincare applications.

## 4. Materials and Methods

### 4.1. Chemicals

2,2-Azino-bis-3-ethylbenzothiazoline-6-sulphonic acid (7 mM ABTS solution; Merck KGaA, Darmstadt, Germany), 2,2-diphenyl-1-picrylhydrazyl (DPPH; Merck KGaA, Darmstadt, Germany), 2′,7′-dichlorodihydrofluorescein diacetate (H_2_DCFDA; Merck KGaA, Darmstadt, Germany), acetic acid (CH_3_COOH; ≥99 Warchem, Zakret, Poland), antibiotics (100 U/mL penicillin and 1000 µg/mL streptomycin; Genos, Łódź, Poland), sterile distilled water (H_2_O; Ultrapure Millipore Direct-Q^®^ 3UV-R; Merck, KGaA, Darmstadt, Germany), DMEM (Dulbecco’s Modification of Eagle’s Medium; VWR International, Radnor, PA, USA), ethanol (C_2_H_5_OH; 96%; Warchem, Zakret, Poland), Fetal Bovine Serum (FBS; Biological Industries, Genos, Lodz, Poland), formic acid (MS-grade acetonitrile; Sigma-Aldrich, St. Louis, MO, USA), HRP conjugate (horseradish peroxidase; Elabscience, Houston, TX, USA), hydrogen peroxide (H_2_O_2_; Warchem, Zakret, Poland), LB Agar (Argenta, Poznań, Poland), methanol (CH_3_OH; Warchem, Zakret, Poland), MRS Agar (Argenta, Poznań, Poland), Neutral Red solution (NR; 0.33%; Sigma-Aldrich, Poznan, Poland), phosphate-buffered saline (PBS; pH 7.00 ± 0.05; Genos, Łódź, Poland), potassium persulfate (2,4 mM; Warchem, Zakret, Poland), resazurin sodium salt (RES; Sigma-Aldrich, Poznan, Poland), Substrate Reagent (3,3′,5,5′-tetramethylbenzidine; Elabscience, TX, USA), Stop Solution (sulfuric acid solution; Elabscience, TX, USA), RIPA (4-nonylphenol; ethoxylated) buffer (EURx; Gdansk, Poland), trypsin–EDTA solution (Sigma-Aldrich, Poznan, Poland) and Trypton Soy Agar (Argenta, Poznań, Poland) were used in the study.

### 4.2. Plant Material, Extraction and Fermentation Procedure

The dried plant material was sourced from Naturalny Sklep (Lublin, Poland) and comprised apple leaves (*Malus domestica*, cv. Gala), apricot leaves (*Prunus armeniaca*, cv. Early Orange) and cherry leaves (*Prunus cerasus*, cv. Nefris). According to the supplier’s documentation, apple, apricot and cherry leaves were assigned voucher numbers 20240915, 20240325 and 20240702, respectively. Three extract types were obtained from each plant material: a non-fermented aqueous extract and extracts fermented with kombucha for 10 and 20 days. The starter culture of the kombucha tea fungus (SCOBY) was purchased from a commercial supplier (Nasza Przyszłość, Tapin, Poland). Extraction was carried out using a combination of magnetic stirring and ultrasound-assisted extraction, in line with the procedure described by Nizioł-Łukaszewska et al. [21]. For each aqueous extract, 5 g of *M. domestica*, *P. armeniaca* or *P. cerasus* leaves were suspended in 100 mL of distilled water. The mixtures were stirred on a magnetic stirrer (Chemland, Stargard, Poland) for 24 h, followed by 0.5 h of sonication. During extraction, the temperature remained within 23.5–26.1 °C. The resulting extracts were filtered under vacuum (Aga Labor, Warsaw, Poland) and stored at 4 °C until further use. Extraction yields were 5.66% for MDWE, 5.55% for PAWE and 5.49% for PCWE. Subsequently, the aqueous extracts were subjected to kombucha fermentation. Aliquots of 200 mL of each extract were transferred into separate sterile 1000 mL beakers. Each extract was supplemented with sucrose to a final concentration of 10.0% (*w*/*v*) (20 g per beaker). Fermentation was initiated by adding 10.0% (*v*/*v*) of liquid culture of kombucha (20 mL) and a symbiotic culture of bacteria and yeast (SCOBY) pellicle (approximately 5 g). The mixtures were then incubated for 10 or 20 days at room temperature (approximately 25 °C), protected from direct sunlight. Both extracts and ferments from *M. domestica*, *P. armeniaca* and *P. cerasus* leaves were then concentrated at 25 °C using a concentrator (Eppendorf Concentrator Plus, Merck KGaA, Darmstadt, Germany) to obtain stock solutions at 5 mg/mL. These stocks were further diluted in the appropriate solvent (water, PBS or cell culture medium) depending on the requirements of the individual assays.

### 4.3. Determination of Biologically Active Compounds Using Chromatographic Analysis

Analytical-grade standards and eluents were sourced from Sigma-Aldrich (St. Louis, MO, USA). Compound separation was conducted using an Infinity II Series ultra-high-performance liquid chromatography (UHPLC) system (Agilent Technologies, Santa Clara, CA, USA) equipped with a diode array detector (DAD) and an Agilent 6224 electrospray ionization time-of-flight mass spectrometer (ESI-TOF MS). Chromatographic separation was achieved on a Kinetex C18 reversed-phase column (100 Å, 150 mm × 2.1 mm, 1.7 µm particle size; Phenomenex, Torrance, CA, USA). The chromatographic method parameters, including mobile phase composition and gradient programme, were applied as previously reported [66]. UV-Vis detection was performed across a spectral range of 200–600 nm. The mass spectrometric analysis was carried out under the following ESI conditions: drying gas temperature at 325 °C with a flow rate of 8 L/min, nebulizer pressure at 30 psi, capillary voltage at 3500 V, fragmentor voltage set to 220 V, and skimmer voltage maintained at 65 V.

### 4.4. Assessment of Antioxidant Activity

#### 4.4.1. DPPH Radical Scavenging Activity Assay

The antioxidant capacity of the analyzed extracts and ferments was determined using the DPPH free radical scavenging method [67]. Test samples were prepared in concentrations of 25–250 µg/mL. Then, 100 µL of the prepared samples were transferred into the wells of 96-well microplate. Afterwards, 100 µL of a 4 mM DPPH methanolic solution was added to each well and thoroughly mixed. Ascorbic acid (AA; Warchem, Zakręt, Poland) and Trolox (TX; Merck KGaA, Darmstadt, Germany) at a concentration of 100 µg/mL were used as positive controls. The absorbance was recorded at 517 nm every 5 min over a 30 min incubation period using a UV–VIS Filter Max spectrophotometer (Thermo Fisher Scientific, Waltham, MA, USA). The antioxidant activity values presented in this study were calculated using the absorbance measured after 20 min, as this time point corresponded to the maximum and stable radical scavenging effect observed for the tested samples and no substantial changes in absorbance were observed thereafter. A mixture of 100 µL of the corresponding solvent (water) and 100 µL of 4 mM DPPH solution, without the tested sample, served as the control. All measurements were performed in triplicate to ensure reproducibility.

The radical scavenging percentage was calculated from the following equation:

(1) $$\%\;DPPH\;scavenging=\frac{Abs\;control-Abs\;sample}{Abs\;control}\times 100$$

where Abs sample is the absorbance of the sample; Abs control is the absorbance of the control sample.

#### 4.4.2. ABTS^+^ Radical Scavenging Assay

The antioxidant potential of the obtained extracts and ferments was also evaluated using the ABTS^+^ radical cation decolorization assay according to Re et al. [68]. To generate ABTS^+^ radicals, equal volumes of 7 mM ABTS solution and 2.4 mM potassium persulfate were mixed and allowed to react at room temperature in the dark for at least 14 h. The resulting solution was subsequently diluted with methanol until its absorbance reached approximately 1.0 at 734 nm. For the assay, sample solutions were prepared at concentrations of 25–250 µg/mL. Ascorbic acid (AA; Warchem, Zakręt, Poland) and Trolox (TX; Merck KGaA, Darmstadt, Germany) at a concentration of 100 µg/mL were used as positive controls. A volume of 1 mL of each sample was combined with 1 mL of the working ABTS^+^ solution, mixed thoroughly, and the absorbance was measured at 734 nm using an Aquamate Helion UV/VIS spectrophotometer (Thermo Fisher Scientific, Waltham, MA, USA). The control consisted of a mixture containing 1 mL of the ABTS^+^ solution and 1 mL of methanol. All measurements were performed in triplicate.

The ABTS^+^ radical scavenging activity was expressed as a percentage inhibition, calculated according to the equation:

(2) $$\%\;ABTS\;scavenging=\left(1-\left(\frac{Abs\;sample}{Abs\;control}\right)\right)\times 100$$

where Abs sample is the absorbance of the sample; Abs control is the absorbance of the control sample.

#### 4.4.3. Detection of Intracellular Levels of Reactive Oxygen Species (ROS)

The antioxidant potential of the examined samples was further evaluated by monitoring the intracellular level of reactive oxygen species (ROS) in human dermal fibroblasts (HDFs) and keratinocytes (HaCaT) according to Wang and Joseph [69] with slight modifications. The analysis was performed using the fluorogenic probe 2′,7′-dichlorodihydrofluorescein diacetate (H_2_DCFDA; Sigma-Aldrich, St. Louis, MO, USA). Both cell types were seeded separately in black 96-well plates at a density of 1 × 10^4^ cells per well and maintained in Dulbecco’s Modified Eagle Medium (DMEM) at 37 °C for 24 h. After incubation, the culture medium was removed and replaced with various concentrations (25–250 µg/mL) of the tested leaf extracts prepared in DMEM. The cells were then incubated for an additional 24 h under standard conditions. Following this exposure period, the extracts were aspirated, and cells were loaded with 10 µM H_2_DCFDA solution in serum-free DMEM. Oxidative stress was induced by adding hydrogen peroxide (H_2_O_2_; Merck KGaA, Darmstadt, Germany) to a final concentration of 500 µM for 60 min. Untreated cells served as the negative control, while cells exposed only to H_2_O_2_ (500 µM) were used as the positive control. After 60 min of incubation with the probe, the intracellular ROS level was quantified by measuring fluorescence intensity using a microplate reader (FilterMax F5, Thermo Fisher Scientific, Waltham, MA, USA) at excitation and emission wavelengths of 485 nm and 530 nm, respectively. Each concentration of extract was analyzed in triplicate across three independent experiments to ensure reproducibility of results. The results are presented as fluorescence intensity.

### 4.5. Assessment of Cytotoxicity on Skin Cells In Vitro

#### 4.5.1. Cell Culture

Cytotoxicity of the investigated extracts and ferments was examined using two skin-derived human cell lines: human dermal fibroblasts (HDFs) and keratinocytes (HaCaT). Both lines used were purchased from CLS Cell Lines Service (Eppelheim, Germany). The cells were cultured in Dulbecco’s Modified Eagle Medium (DMEM) supplemented with sodium pyruvate, L-glutamine, glucose (4.5 g/L), 10% fetal bovine serum (FBS) and 1% antibiotics (1000 U/mL penicillin and 1000 µg/mL streptomycin). Anti-inflammatory activity was evaluated using THP-1 monocytes purchased from Merck (Merck KGaA, Darmstadt, Germany). These cells were cultured in RPMI-1640 medium supplemented with 10% fetal bovine serum (FBS) and 1% penicillin/streptomycin solution containing 100 U/mL penicillin and 1000 µg/mL streptomycin. All cells were kept under standard culture conditions (at 37 °C in a humidified 5% CO_2_ atmosphere). When cell cultures reached approximately 70–80% confluency, they were detached with 0.25% trypsin–EDTA. Cells were subsequently seeded into 96-well plates at a density of 1 × 10^4^ cells per well.

#### 4.5.2. Alamar Blue Assay

Mitochondrial activity was examined using the Alamar Blue method. A fresh working solution of resazurin (60 µM) was prepared in complete culture medium and applied to wells containing cells preincubated with the tested compounds at concentrations ranging from 25 to 250 µg/mL. Untreated HDF or HaCaT cells maintained solely in DMEM served as negative controls. The plate was then incubated for 2 h at 37 °C in a humid atmosphere containing 5% CO_2_. After this time, fluorescence was recorded with a microplate reader (Thermo Fisher Scientific, Waltham, MA, USA) at an excitation wavelength of 570 nm. Each experimental condition was analyzed in triplicate in at least three independent experiments. Fluorescence intensities were normalized to the corresponding control values and expressed as percentages to facilitate comparison between sample groups using the formula below. The procedure followed the general principles described in reference [70].

(3) $$cell\;viability\;[\%]=\frac{Abs\;sample}{Abs\;control}\times 100$$

where Abs sample is absorbance of the sample; Abs control is absorbance of the control sample.

#### 4.5.3. Neutral Red Assay

To assess lysosomal activity as an indicator of cell viability, the Neutral Red uptake test was performed. After exposure to the tested extracts and ferments (at concentrations ranging from 25 to 250 µg/mL), cells were incubated with Neutral Red diluted in DMEM, enabling selective accumulation of the dye inside lysosomes of viable cells. After a 2 h incubation period at 37 °C in a humid atmosphere containing 5% CO_2_, excess dye was removed by washing the wells with phosphate-buffered saline (PBS). In order to release the absorbed dye, a decolorizing solution consisting of ethanol, acetic acid, and water in a ratio of 50%:1%:49% was added. The prepared plates were shaken on an orbital shaker for 15 min, and absorbance was measured at 540 nm using a microplate reader (Thermo Fisher Scientific, Waltham, MA, USA). All measurements were conducted in triplicate, and the results were expressed relative to untreated control cells according to the procedure described by Borenfreund and Puerner with some modifications [71].

(4) $$cell\;viability\;[\%]=\frac{Abs\;sample}{Abs\;control}\times 100$$

where Abs sample is absorbance of the sample; Abs control is absorbance of the control sample.

### 4.6. Assessment of Antimicrobial Activity

#### 4.6.1. Disc Diffusion Assay

The influence of the analyzed fermented and unfermented leaf extracts on the growth of pathogenic bacteria was examined using the disc diffusion assay, following the procedure previously reported by Zagórska-Dziok et al. [2]. The bacterial strains were obtained from the American Type Culture Collection (ATCC, Manassas, VA, USA). Antimicrobial activity was evaluated against *Staphylococcus aureus* ATCC BAA-2312, *Staphylococcus capitis* ATCC^®^ 146™, *Staphylococcus epidermidis* ATCC^®^ 49134™, *Bacillus subtilis* ATCC^®^ 19659, *Micrococcus luteus* ATCC^®^ 10240™, *Yersinia enterocolitica* ATCC^®^ 27729 and *Pseudomonas aeruginosa* ATCC^®^ 35032. Initially, 10 mL of agar medium suitable for each bacterial species were dispensed into sterile Petri dishes. The media employed in the study included Chapman agar, tryptone soy agar, nutrient agar, LB agar and MRS agar (Argenta, Poznań, Poland). After solidification, the plates were inoculated with the respective bacterial suspensions, adjusted to a density of 5 × 10^7^ colony-forming units (CFU)/mL. Sterile filter paper discs were then impregnated with the tested extracts at concentrations of 250 and 500 µg/mL. Prior to use, the extract solutions were sterilized by passage through 0.22 µm membrane filters. The loaded discs were placed on the surface of the inoculated agar plates. Discs soaked in sterile deionized water served as negative controls. As positive controls, antibiotics appropriate for each microorganism were applied: vancomycin for *S. aureus*, *S. epidermidis*, *S. capitis* and *B. subtilis*; erythromycin for *M. luteus*; and ciprofloxacin for *Y. enterocolitica* and *P. aeruginosa.* The prepared plates were incubated at 35 ± 2 °C for 24 h. Thereafter, antimicrobial activity was quantified by measuring the diameter of the zones of growth inhibition around the discs.

#### 4.6.2. Determination of Minimum Inhibitory Concentrations (MICs)

The antimicrobial properties of the samples (extracts and ferments) were further evaluated by determining their minimum inhibitory concentrations (MICs). To this end, the extracts were evaporated to dryness and subsequently reconstituted in sterile distilled water to obtain stock solutions at 10 mg/mL. MIC values were determined by a broth microdilution method using p-iodonitrotetrazolium violet (INT, Merck KGaA, Darmstadt, Germany) as a growth indicator, following the procedure described by Eloff [72]. Serial dilutions in broth were prepared to yield final extract concentrations ranging from 25 to 2000 μg/mL. The resulting dilutions (in triplicate) were dispensed into flat-bottom 96-well microplates, after which a suspension of the respective bacterial strain (5 × 10^4^ colony-forming units (CFU) per well) was added. For each microorganism, a sterility control, an antibiotic control and a growth control were included. The plates were incubated for 24 h at 37 °C, then 40 μL of INT solution (0.4 mg/mL) was added to each well and incubation continued for 30 min. The MIC was defined as the lowest concentration of the extract that prevented visible growth of the tested bacterial strain. All measurements were carried out in three independent experiments.

### 4.7. Assessment of Anti-Inflammatory Activity

To investigate the anti-inflammatory activity of the extracts and ferments from *M. domestica*, *P. armeniaca* and *P. cerasus* leaves, the concentrations of TNF-α, IL-1β and IL-6 were determined in THP-1 human monocytic cells stimulated with bacterial lipopolysaccharide (LPS, 10 µg/mL; *Escherichia coli* O111:B4) for 24 h. In parallel, cells seeded in 6-well plates were exposed to the tested extracts at a concentration of 250 µg/mL. Diclofenac at 10 µg/mL was included as a reference compound in the assay. Following 24 h incubation with LPS and the respective samples, DMEM was aspirated from the wells, which were subsequently rinsed with PBS. Next, 150 µL of RIPA buffer was added to each well to lyse the cells. The resulting lysates were analyzed using commercial ELISA kits (Elabscience Biotechnology Inc., Houston, TX, USA) following the manufacturer’s protocol. Absorbance was recorded at 450 nm using a microplate reader (FilterMax F5, Thermo Fisher Scientific, Waltham, MA, USA). Cells neither exposed to LPS nor treated with extracts served as the negative control (NC), whereas LPS-stimulated cells not receiving any extract treatment were used as the positive control (PC). Results are presented as fold change relative to the negative control NC (cells untreated with either the test compounds or LPS).

### 4.8. Assessment of Anti-Ageing Activity Through Inhibition of Collagenase, Elastase and Hyaluronidase

In this study, the effects extracts and ferments obtained from the leaves of the three studied plant species on collagenase, elastase and hyaluronidase levels were examined in human dermal fibroblasts (HDFs). The extracts were tested at a concentration of 250 µg/mL. Fibroblasts were seeded into flat-bottom 6-well plates at a density of 1 × 10^4^ cells per well. After 24 h exposure to the respective extracts, the cells were washed twice with sterile PBS and lysed by adding 150 µL of RIPA lysis buffer to each well. The resulting cell lysates were used to determine the intracellular levels of the individual enzymes with commercially available ELISA kits (Human COL2α1 ELISA kit, Human Ne/ELA2 ELISA kit, Human HAase (Hyaluronidase) ELISA Kit; Elabscience Biotechnology Inc., Houston, TX, USA), following the manufacturer’s instructions. Succinyl-alanyl-alanyl-prolyl-valine-chloromethyl ketone (SPCK, Adooq Bioscience, Irvine, CA, USA) served as the reference inhibitor for elastase, 1,10-phenanthroline (Abcam, Cambridge, UK) for collagenase, and tannic acid (Merck KGaA, Darmstadt, Germany) for hyaluronidase. Absorbance was recorded at 450 nm using a microplate reader (FilterMax F5, Thermo Fisher Scientific, Waltham, MA, USA). The assays were carried out in three independent experiments, with each sample analyzed in duplicate.

### 4.9. Statistical Analysis

Statistical analysis began with verification of the normality of data distribution using the Shapiro–Wilk test. The results for the examined parameters are expressed as mean ± standard deviation (SD). Subsequently, statistical significance was evaluated by two-way analysis of variance (ANOVA), followed by Dunnett’s post hoc test for multiple comparisons. Differences were considered statistically significant at **** *p* < 0.0001, *** *p* < 0.001, ** *p* < 0.01 and * *p* < 0.05 versus the control group. All statistical calculations were carried out using GraphPad Prism 8.4.3 (GraphPad Software, Inc., San Diego, CA, USA).

## 5. Conclusions

This study demonstrated for the first time that leaves of *Malus domestica*, *Prunus armeniaca*, and *Prunus cerasus*, considered low-value agro-waste materials, can be successfully transformed through kombucha fermentation into biologically active preparations with potential applications in cosmetology and dermatology. The obtained results showed that both aqueous extracts and their fermented counterparts are rich in phenolic compounds, particularly flavonoids and phenolic acids, while the fermentation process significantly modified their phytochemical profiles and enhanced several biological properties.

Kombucha fermentation increased the antioxidant potential of the investigated samples in chemical assays and effectively reduced intracellular ROS levels in keratinocytes and fibroblasts, confirming the biological relevance of the observed activity. In addition, the ferments demonstrated improved antimicrobial, anti-inflammatory, and anti-ageing effects compared with extracts, while maintaining satisfactory cytocompatibility within the tested concentration range. The magnitude of these effects depended on the plant species and fermentation time, indicating that optimization of the biotransformation process is essential for obtaining preparations with desired biological properties.

Importantly, this work expands current knowledge regarding the valorization of orchard waste biomass and highlights the potential of SCOBY-mediated fermentation as an environmentally friendly strategy for the production of multifunctional plant-derived ingredients. The observed enhancement of biological activity after fermentation suggests that microbial biotransformation may improve the bioavailability and functional properties of naturally occurring phenolic compounds.

Overall, the present findings provide a scientific basis for the further development of fermented fruit tree leaf preparations as sustainable, bio-based ingredients for protective, anti-ageing, and regenerative skincare formulations. At the same time, the study underlines the need for further investigations using advanced skin models and in vivo approaches to better understand the mechanisms underlying the observed effects and to confirm their efficacy and safety under physiological conditions.

## Figures and Tables

**Table 1:** Results of the quantification of the main secondary metabolites identified in *M. domestica* extract (MDE) and in ferments after 10 (MDF10) and 20 (MDF20) days of fermentation. Different letters indicate statistically significant differences.

Compound	MDE (µg/mL)	MDF10 (µg/mL)	MDF20 (µg/mL)
Gallic acid *	0.73 ± 0.06 ^b^	5.43 ± 0.46 ^a^	5.49 ± 0.32 ^a^
Gentisic acid hexoside	10.55 ± 1.02 ^a^	9.07 ± 0.62 ^a^	10.91 ± 1.11 ^a^
Protocatechuic acid *	10.81 ± 0.36 ^a^	10.11 ± 0.29 ^a^	10.04 ± 0.31 ^a^
Chlorogenic acids *	21.68 ± 0.52 ^b^	36.10 ± 2.01 ^a^	37.82 ± 2.81 ^a^
*p*-Coumaroylquinic acids	23.42 ± 1.12 ^b^	29.48 ± 1.73 ^a^	32.33 ± 1.93 ^a^
Caffeic acid *	9.08 ± 0.52 ^b^	12.44 ± 0.51 ^a^	12.66 ± 0.21 ^a^
Quercetin pentosides *m*/*z*-H = 433	3.11 ± 0.18 ^a^	4.03 ± 0.28 ^a^	3.52 ± 0.12 ^a^
Quercetin hexosides *m*/*z*-H = 463	6.29 ± 0.41 ^b^	8.19 ± 0.54 ^a^	7.59 ± 0.64 ^a^
Kaempferol-3-*O*-glucoside *	1.78 ± 0.10 ^a^	1.82 ± 0.12 ^a^	1.69 ± 0.13 ^a^
Quercetin 3-rhamnoside *	32.94 ± 1.80 ^b^	34.66 ± 1.92 ^b^	38.87 ± 2.01 ^a^
Phloridzin *	1.36 ± 0.01 ^a^	1.24 ± 0.02 ^a^	1.41 ± 0.01 ^a^
Eriodictyol *	2.11 ± 0.18 ^c^	6.47 ± 0.34 ^b^	10.58 ± 0.63 ^a^

*—the identity was confirmed by comparison with a standard.

**Table 2:** Results of the quantification of the main secondary metabolites identified in *P. armeniaca* extract (PAE) and in ferments after 10 (PAF10) and 20 (PAF20) days of fermentation. Different letters indicate statistically significant differences.

Compound	PAE (µg/mL)	PAF10 (µg/mL)	PAF20 (µg/mL)
Gallic acid *	0.02 ± 0.00 ^b^	2.07 ± 0.11 ^a^	2.79 ± 0.12 ^a^
Protocatechuic acid *	2.31 ± 0.16 ^b^	2.34 ± 0.09 ^b^	3.34 ± 0.19 ^a^
Chlorogenic acids *	723.9 ± 12.8 ^b^	718.10 ± 21.4 ^b^	769.82 ± 20.8 ^a^
*p*-Coumaroylquinic acids	88.42 ± 4.02 ^b^	85.48 ± 3.76 ^b^	106.3 ± 6.87 ^a^
Caffeic acid *	7.08 ± 0.32 ^a^	7.21± 0.41 ^a^	6.96 ± 0.37 ^a^
*p*-Coumaric acid glucosides	8.27 ± 0.34 ^b^	9.03 ± 0.41 ^a,b^	10.51 ± 0.62 ^a^
Feruloylquinic acids	11.91 ± 1.01 ^a^	12.04 ± 0.65 ^a^	12.38 ± 0.94 ^a^
Quercetin diglycosides *m*/*z*-H = 609	138.2 ± 7.21 ^b^	136.8 ± 6.33 ^b^	151.6 ± 8.45 ^a^
Quercetin 3-*O*-glucoside *	9.89 ± 0.31 ^b^	10.11 ± 0.48 ^b^	14.5 ± 0.49 ^a^
Quercetin acetyl hexoside	2.01 ± 0.12 ^a^	1.87 ± 0.13 ^a^	1.99 ± 0.18 ^a^
Kaempferol-3-*O*-rutinoside *	11.58 ± 0.96 ^a^	11.67 ± 0.57 ^a^	13.02 ± 1.03 ^a^
Kaempferol-3-*O*-glucoside *	2.61 ± 0.10 ^b^	2.36 ± 0.12 ^b^	3.27 ± 0.21 ^a^

*—the identity was confirmed by comparison with a standard.

**Table 3:** Results of the quantification of the main secondary metabolites identified in *P. cerasus* extract (PCE) and in ferments after 10 (PCF10) and 20 (PCF20) days of fermentation. Different letters indicate statistically significant differences.

Compound	PCE (µg/mL)	PCF10 (µg/mL)	PCF20 (µg/mL)
Gallic acid *	0.54 ± 0.01 ^b^	3.33 ± 0.11 ^a^	3.27 ± 0.12 ^a^
Hydroxybenzoic acid glucosides	7.35 ± 0.59 ^a^	6.71 ± 0.25 ^a,b^	6.06 ± 0.42 ^b^
Gentisic acid hexoside	5.18 ± 0.32 ^a^	5.49 ± 0.33 ^a^	5.50 ± 0.62 ^a^
Protocatechuic acid	4.99 ± 0.22 ^a^	4.60 ± 0.15 ^a, b^	4.25 ± 0.21 ^b^
Chlorogenic acids *	23.21 ± 1.01 ^a^	23.48 ± 0.68 ^a^	21.18 ± 0.61 ^b^
*p*-Coumaroylquinic acids	32.01 ± 0.80 ^a^	34.96 ± 1.03 ^a^	27.90 ± 1.14 ^b^
*p*-Coumaric acid glucosides	58.29 ± 6.18 ^a^	55.73 ± 4.41 ^a^	49.04 ± 2.34 ^b^
Caffeic acid *	5.69 ± 0.31 ^a^	4.71 ± 0.02 ^b^	4.34 ± 0.06 ^b^
Feruloylquinic acids	2.23 ± 0.11 ^a^	2.30 ± 0.13 ^a^	2.37 ± 0.16 ^a^
Quercetin derivative *m*/*z*-H = 771	2.10 ± 0.07 ^b^	7.04 ± 0.32 ^a^	6.11 ± 0.31 ^a^
Quercetin derivative *m*/*z*-H = 775	3.36 ± 0.34 ^b^	12.11 ± 1.02 ^a^	10.43 ± 0.95 ^a^
Isorhamnetin derivative *m*/*z*-H = 785	3.54 ± 0.17 ^b^	20.52 ± 1.08 ^a^	17.88 ± 1.20
Quercetin 3-*O*-rutinoside *	1.60 ± 0.09 ^c^	11.71 ± 1.03 ^a^	7.65 ± 0.42 ^b^
Quercetin 3-*O*-glucoside *	1.18 ± 0.11 ^b^	1.63 ± 0.08 ^a^	1.27 ± 0.03 ^b^
Kaempferol-3-*O*-rutinoside *	2.72 ± 0.05 ^c^	47.01 ± 3.11 ^a^	25.23 ± 1.15 ^b^
Isorhamnetin-3-*O*-rutinoside *	5.01 ± 0.23 ^c^	36.28 ± 1.01 ^a^	29.43 ± 1.04 ^b^
Kaempferol-3-*O*-glucoside *	3.05 ± 0.12 ^a,b^	3.58 ± 0.11 ^a^	2.89 ± 0.22 ^b^
Isorhamnetin hexoside	3.99 ± 0.12 ^a^	4.19 ± 0.13 ^a^	4.05 ± 0.15 ^a^

*—the identity was confirmed by comparison with a standard.

**Table 4:** Antimicrobial activity of the tested *M. domestica*, *P. armeniaca*, and *P. cerasus* leaf extracts and ferments expressed as the diameter of the average inhibition zone (mm).

Bacteria	Plant Species	Minimum Inhibitory Concentration [µg/mL]					
		Extract		10-Day Ferment		20-Day Ferment	
		250	500	250	500	250	500
*Staphylococcus aureus*	*M. domestica*	7	9	9	9	11	11
	*P. armeniaca*	6	10	9	15	12	16
	*P. cerasus*	12	9	13	16	14	16
*Staphylococcus epidermidis*	*M. domestica*	7	11	8	11	11	16
	*P. armeniaca*	6	9	10	16	12	18
	*P. cerasus*	nd	nd	8	10	14	16
*Bacillus subtilis*	*M. domestica*	7	10	8	10	9	11
	*P. armeniaca*	5	7	6	10	12	13
	*P. cerasus*	nd	nd	4	5	10	10
*Staphylococcus capitis*	*M. domestica*	10	9	12	13	15	18
	*P. armeniaca*	4	7	8	9	12	12
	*P. cerasus*	9	7	9	9	10	12
*Micrococcus luteus*	*M. domestica*	nd	nd	5	5	8	11
	*P. armeniaca*	nd	5	7	8	11	15
	*P. cerasus*	nd	nd	nd	nd	5	8
*Yersinia enterocolitica*	*M. domestica*	nd	nd	6	7	10	12
	*P. armeniaca*	4	7	8	8	11	18
	*P. cerasus*	nd	nd	5	7	9	9
*Pseudomonas aeruginosa*	*M. domestica*	6	7	8	10	12	19
	*P. armeniaca*	5	7	11	15	15	18
	*P. cerasus*	5	7	7	11	12	14

nd—not detected.

**Table 5:** Minimum inhibitory concentrations (MICs) of *M. domestica*, *P. armeniaca*, and *P. cerasus* leaf extracts and ferments against the tested bacteria.

Bacteria	Plant Species	Minimum Inhibitory Concentration [µg/mL]		
		Extract	10-Day Ferment	20-Day Ferment
*Staphylococcus aureus*	*M. domestica*	350	250	150
	*P. armeniaca*	350	300	250
	*P. cerasus*	300	250	200
*Staphylococcus epidermidis*	*M. domestica*	250	200	150
	*P. armeniaca*	350	250	150
	*P. cerasus*	nd	500	400
*Bacillus subtilis*	*M. domestica*	350	250	200
	*P. armeniaca*	400	300	200
	*P. cerasus*	nd	nd	450
*Staphylococcus capitis*	*M. domestica*	300	200	150
	*P. armeniaca*	400	200	150
	*P. cerasus*	400	250	150
*Micrococcus luteus*	*M. domestica*	nd	450	300
	*P. armeniaca*	600	400	300
	*P. cerasus*	nd	nd	500
*Yersinia enterocolitica*	*M. domestica*	nd	800	500
	*P. armeniaca*	400	200	100
	*P. cerasus*	nd	600	500
*Pseudomonas aeruginosa*	*M. domestica*	350	250	200
	*P. armeniaca*	400	300	200
	*P. cerasus*	450	400	350

nd—not detected.

## Data Availability

The original contributions presented in this study are included in the article/Supplementary Material. Further inquiries can be directed to the corresponding author.

## Supplementary Materials

The following supporting information can be downloaded at https://www.mdpi.com/article/10.3390/ijms27125328/s1.

## Abbreviations

The following abbreviations are used in this manuscript:

AA	ascorbic acid
AB	Alamar Blue Assay
ABTS	2,2′-azino-bis(3-ethylbenzothiazoline-6-sulfonic acid)
AHA	alpha hydroxy acids
CFU	colony-forming units
CO_2_	carbon dioxide
COL	collagenase
COX-2	cyclooxygenase 2
D	diclofenac
DAD	diode array detector
DCF	2′,7′-dichlorofluorescein
DMEM	Dulbecco’s modified eagle medium
DPPH	1,1-diphenyl-2-picrylhydrazyl
E	extract
ELA	elastase
ELISA	enzyme-linked immunosorbent assay
ESI/TOF	Electrospray Ionization/Time-of-Flight
F10	10-day ferment
F20	20-day ferment
FBS	fetal bovine serum
FRAP	ferric reducing antioxidant power
H_2_DCFDA	2′,7′-dichlorodihydrofluorescein diacetate
H_2_O_2_	hydrogen peroxide
HAase	hyaluronidase
HaCaT	human keratinocyte cell line
HDF	human dermal fibroblasts
IL-10	interleukin 10
IL-1β	interleukin-1 beta
IL-6	interleukin 6
INT	p-iodonitrotetrazolium violet
iNOS	inducible nitric oxide synthase
LPS	lipopolysaccharide
MIC	minimum inhibitory concentration
NC	negative control
NF-κB	nuclear factor kappa B
NO	nitric oxide
NR	Neutral Red Assay
PBS	phosphate-buffered saline
PC	positive control
ROS	reactive oxygen species
SCOBY	symbiotic culture of bacteria and yeast
THP-1	human acute monocytic leukemia cell line
TNF-α	tumour necrosis factor alpha
TPTZ	2,4,6-tripyridyl-s-triazine
TX	Trolox
UHPLC	ultra-high-performance liquid chromatography
UV-VIS	ultraviolet–visible spectroscopy
