# Supplementary material for: Kombucha-Mediated Fermentation Enhances Antioxidant, Anti-Inflammatory, Anti-Ageing and Antimicrobial Properties of Fruit Tree Leaf Agro-Waste Extracts from Malus domestica, Prunus armeniaca and Prunus cerasus
Source: Int J Mol Sci. 2026 Jun 12;27(12):5328. doi: 10.3390/ijms27125328 (PMC13299957; doi:10.3390/ijms27125328)
Supplement: Supplementary file 1 [file ijms-27-05328-s001.zip › ijms-4337558-supplementary.pdf]

International Journal of *Molecular Sciences*

Article

Kombucha-Mediated Fermentation Enhances Antioxidant, Anti-Inflammatory, Anti-Aging and Antimicrobial Properties of Fruit Tree Leaf Agro-Waste Extracts from *Malus domestica*, *Prunus armeniaca* and *Prunus cerasus*

Martyna Zagórska-Dziok[1*], Aleksandra Ziemlewska[1], Zofia Nizioł-Łukaszewska[1], Agnieszka Mokrzyńska[1], Magdalena Wójciak[2], Justyna Zagórska[3], Ireneusz Sowa[2]

[1] Department of Technology of Cosmetic and Pharmaceutical Products, Medical College, University of Information Technology and Management in Rzeszow, Sucharskiego 2, 35-225 Rzeszow, Poland; mzagorska@wsiz.edu.pl; aziemlewska@wsiz.edu.pl; zniziol@wsiz.edu.pl; amokrzynska@wsiz.edu.pl
[2] Department of Analytical Chemistry, Medical University of Lublin, Aleje Raclawickie 1, 20-059 Lublin, Poland; magdalena.wojciak@umlub.pl; ireneusz.sowa@umlub.pl
[3] Department of Food and Nutrition, Medical University of Lublin, 4a Chodzki Str., 20-093 Lublin, Poland; justyna.zagorska@umlub.pl

* Correspondence: mzagorska@wsiz.edu.pl

Table S1. Mass data used to identify the main constituents of *Prunus cerasus* leaf extract.

Rt (min.)	m/z-H/ (Fragments)	Δ ppm	Formula	Compound
1.73	195.05147	2.26	$C_6H_{12}O_7$	Gluconic Acid
1.83	191.05674	3.27	$C_7H_{12}O_6$	Quinic acid
4.73	169.01484 (125)	3.49	$C_7H_6O_5$	Gallic acid*
6.62	299.07655 (137)	-2.30	$C_{13}H_{16}O_8$	Hydroxybenzoic acid glucoside
7.75	315.07342	4.00	$C_{13}H_{16}O_9$	Gentisic acid hexoside
8.65	153.01975	2.71	$C_7H_6O_4$	Protocatechuic acid*
9.57	329.08903 (167)	3.71	$C_{14}H_{18}O_9$	Vanillic acid glucoside
11.25	353.08804 (179,191)	0.66	$C_{16}H_{18}O_9$	Neochlorogenic acid*
11.98	343.10280 (181)	-1.91	$C_{15}H_{20}O_9$	Methyl vanillate glucoside
13.05	299.07821 (137)	3.23	$C_{13}H_{16}O_8$	Hydroxybenzoic acid glucoside
13.61	337.09391 (163, 191,173)	3.01	$C_{16}H_{18}O_8$	3-*p*-Coumaroylquinic acid
14.33	337.09366 (163, 191,173)	2.27	$C_{16}H_{18}O_8$	3-*p*-Coumaroylquinic acid
14.89	325.09358 (163)	2.11	$C_{15}H_{18}O_8$	*p*-Coumaric acid glucoside
15.14	325.09373 (163)	2.57	$C_{15}H_{18}O_8$	*p*-Coumaric acid glucoside
16.27	353.08856 (191, 179)	2.13	$C_{16}H_{18}O_9$	Chlorogenic acid*
16.58	353.08901 (191, 179)	3.40	$C_{16}H_{18}O_9$	Cryptochlorogenic acid*
17.06	179.03506	0.43	$C_9H_8O_4$	Caffeic acid*
17.63	431.19214	-0.30	$C_{20}H_{32}O_{10}$	unknown
17.93	337.09359 (191,173)	2.07	$C_{16}H_{18}O_8$	4-p-Coumaroylquinic acid
18.66	431.19237	0.23	$C_{20}H_{32}O_{10}$	unknown
19.14	327.10893	1.19	$C_{15}H_{20}O_8$	unknown
19.76	337.09342 (191,173)	1.56	$C_{16}H_{18}O_8$	4-p-Coumaroylquinic acid
20.38	337.09356 (191,173)	1.98	$C_{16}H_{18}O_8$	5-p-Coumaroylquinic acid
21.14	325.09342 (163)	1.62	$C_{15}H_{18}O_8$	p-Coumaric acid glucoside
22.46	367.10447	2.76	$C_{17}H_{20}O_9$	Feruloylquinic acid
22.69	337.09402 (191,173)	3.34	$C_{16}H_{18}O_8$	5-p-Coumaroylquinic acid
24.55	771.20234 (463)	4.41	$C_{33}H_{40}O_{21}$	Quercetin derivative
24.87	755.20196	-2.72	$C_{33}H_{40}O_{20}$	Kaempferol derivative
26.04	165.05503	-4.14	$C_9H_{10}O_3$	unknown
27.47	785.21673	2.73	$C_{34}H_{42}O_{21}$	Isorhamnetin derivative
29.57	433.11539 (271)	3.15	$C_{21}H_{22}O_{10}$	Naringenin-7-O-glucoside*
32.07	609.14709 (300)	1.61	$C_{27}H_{30}O_{16}$	Quercetin 3-O- rutinoside*
33.57	463.08943 (300)	2.65	$C_{21}H_{20}O_{12}$	Quercetin 3-O- glucoside*
38.03	593.15211	1.54	$C_{27}H_{30}O_{15}$	Kaempferol-3-O-rutinoside*
39.05	515.12077 (353)	2.46	$C_{25}H_{24}O_{12}$	3,5-Dicaffeoylquinic acid*
39.16	623.16093	-1.33	$C_{28}H_{32}O_{16}$	Isorhamnetin-3-O-rutinoside*
39.37	447.09457	2.87	$C_{21}H_{20}O_{11}$	Kaempferol-3-O- glucoside*
40.43	477.10584	4.16	$C_{22}H_{22}O_{12}$	Isorhamnetin hexoside
42.96	515.12039 (353)	1.72	$C_{25}H_{24}O_{12}$	4,5-Dicaffeoylquinic acid*

* Identification was confirmed using a standard

Table S2. Mass data used to identify the main constituents of *Prunus armeniaca* leaf extract.

Rt (min.)	m/z-H (Fragments)	Δppm	Formula	Compound
1.73	195.05169	3.39	$C_6H_{12}O_7$	Gluconic Acid
1.80	191.05681	3.64	$C_7H_{12}O_6$	Quinic acid
4.46	371.09702 (197)	-3.63	$C_{16}H_{20}O_{10}$	Syringoylquinic acid
4.80	169.01490 (125)	4.25	$C_7H_6O_5$	Gallic acid*
6.62	371.09741 (197)	-2.58	$C_{16}H_{20}O_{10}$	Syringoylquinic acid
6.68	299.07679 (137)	-1.50	$C_{13}H_{16}O_8$	Hydroxybenzoic acid glucoside
7.44	371.09773 (197)	-1.72	$C_{16}H_{20}O_{10}$	Syringoylquinic acid
7.78	315.07339	3.91	$C_{13}H_{16}O_9$	Dihydroxybenzoic acid hexoside
8.77	153.01961	1.80	$C_7H_6O_4$	Protocatechuic acid*
11.32	353.08796 (179,191)	0.44	$C_{16}H_{18}O_9$	Neochlorogenic acid*
12.60	325.09311	0.67	$C_{15}H_{18}O_8$	*p*-Coumaric acid glucoside
13.72	337.09299 (163, 191,173)	0.29	$C_{16}H_{18}O_8$	3-*p*-Coumaroylquinic acid
14.40	337.09273 (163, 191,173)	-0.48	$C_{16}H_{18}O_8$	3-*p*-Coumaroylquinic acid
14.91	487.14644	1.49	$C_{21}H_{28}O_{13}$	unknown
15.25	325.09334	1.38	$C_{15}H_{18}O_8$	*p*-Coumaric acid glucoside
16.33	353.08917 (191, 179)	3.85	$C_{16}H_{18}O_9$	Chlorogenic acid*
16.60	353.08868 (191, 179)	2.47	$C_{16}H_{18}O_9$	Cryptochlorogenic acid*
17.08	179.03522	1.32	$C_9H_8O_4$	Caffeic acid*
17.97	337.09301 (191,173)	0.35	$C_{16}H_{18}O_8$	4-*p*-Coumaroylquinic acid
19.80	337.09329 (191,173)	1.18	$C_{16}H_{18}O_8$	4-*p*-Coumaroylquinic acid
20.40	337.09398 (191,173)	3.22	$C_{16}H_{18}O_8$	5-*p*-Coumaroylquinic acid
21.61	367.10401	1.51	$C_{17}H_{20}O_9$	Feruloylquinic acid
22.48	367.10422	2.08	$C_{17}H_{20}O_9$	Feruloylquinic acid
22.73	337.09385 (191,173)	2.84	$C_{16}H_{18}O_8$	5-*p*-Coumaroylquinic acid
30.91	609.14692	1.33	$C_{27}H_{30}O_{16}$	Quercetin 7-O- rutinoside*
32.04	609.14680	1.13	$C_{27}H_{30}O_{16}$	Quercetin 3-O- rutinoside*
33.66	463.08921 (301, 300)	2.18	$C_{21}H_{20}O_{12}$	Quercetin 3-O- glucoside*
37.74	505.09861 (463, 300)	-0.30	$C_{23}H_{22}O_{13}$	Quercetin acetyl hexoside
38.09	593.15179	1.00	$C_{27}H_{30}O_{15}$	Kaempferol-3-O-rutinoside*
39.34	447.09438	2.44	$C_{21}H_{20}O_{11}$	Kaempferol-3-O-glucoside*
39.67	447.09444 (300)	2.58	$C_{21}H_{20}O_{11}$	Quercitrin*
41.30	505.09705 (300)	-3.39	$C_{23}H_{22}O_{13}$	Quercetin acetyl hexoside

* Identification was confirmed using standard

Table S3. Mass data used to identify the main constituents of *Malus domestica* leaf extract.

Rt (min.)	m/z-H (Fragments)	Δppm	Formula	Compound
1.75	195.05163	3.08	$C_6H_{12}O_7$	Gluconic acid
1.83	191.05672	3.17	$C_7H_{12}O_6$	Quinic acid
4.80	169.01490 (125)	3.84	$C_7H_6O_5$	Gallic acid
6.50	467.11989 (305)	0.83	$C_{21}H_{24}O_{12}$	(Epi)gallocatechin der
6.71	467.11818 (305)	-2.82	$C_{21}H_{24}O_{12}$	(Epi)gallocatechin der
7.01	331.06574	-4.01	$C_{13}H_{16}O_{10}$	galloylglucose
7.48	305.06563	-3.42	C15H14O7	(Epi)gallocatechin
7.78	315.07291 (153)	2.39	$C_{13}H_{16}O_9$	gentisic acid hexoside
8.75	153.01984	3.30	$C_7H_6O_4$	Protocatechuic acid
9.50	299.07787 (137)	4.86	$C_{13}H_{16}O_8$	Hydroxybenzoic acid glucoside
11.20	353.08857 (179,191)	2.16	$C_{16}H_{18}O_9$	Neochlorogenic acid
13.61	337.09398 (163, 191,173)	3.22	$C_{16}H_{18}O_8$	3-*p*-Coumaroylquinic acid
14.33	337.09352 (163, 191,173)	1.86	$C_{16}H_{18}O_8$	3-*p*-Coumaroylquinic acid
15.20	325.09367	2.39	$C_{15}H_{18}O_8$	*p*-Coumaric acid glucoside
15.63	289.07203	0.92	$C_{15}H_{14}O_6$	Catechin*
16.30	353.08866 (191, 179)	2.41	$C_{16}H_{18}O_9$	Chlorogenic acid
16.58	353.08829 (191, 179)	1.37	$C_{16}H_{18}O_9$	Cryptochlorogenic acid
17.07	179.03548	2.76	$C_9H_8O_4$	Caffeic acid
17.89	337.09401 (191,173)	3.31	$C_{16}H_{18}O_8$	4-p-Coumaroylquinic acid
18.71	431.19078	-3.45	$C_{20}H_{32}O_{10}$	unknown
19.53	289.07276	3.44	$C_{15}H_{14}O_6$	Epicatechin*
19.59	465.10362	-0.49	$C_{21}H_{22}O_{12}$	unknown
19.74	337.09381 (191,173)	2.72	$C_{16}H_{18}O_8$	4-*p*-Coumaroylquinic acid
20.34	337.09344 (191,173)	1.62	$C_{16}H_{18}O_8$	5-*p*-Coumaroylquinic acid
22.71	337.09389 (191,173)	2.95	$C_{16}H_{18}O_8$	5-*p*-Coumaroylquinic acid
32.03	609.14738	2.08	$C_{27}H_{30}O_{16}$	Quercetin 3-*O*- rutinoside*
32.40	463.08951 (301, 300)	2.82	$C_{21}H_{20}O_{12}$	Quercetin 3-*O*- galactoside*
33.53	463.08936 (301, 300)	2.50	$C_{21}H_{20}O_{12}$	Quercetin 3-*O*-glucoside*
36.02	433.07803	0.91	$C_{20}H_{18}O_{11}$	Quercetin pentoside
38.13	433.07839	1.74	$C_{20}H_{18}O_{11}$	Quercetin-3-*O*-arabinoside*
38.85	433.07811	1.09	$C_{20}H_{18}O_{11}$	Quercetin pentoside
39.30	447.09398	1.55	$C_{21}H_{20}O_{11}$	Kaempferol-3-*O*-glucoside*
39.65	447.09429 (300)	2.24	$C_{21}H_{20}O_{11}$	Quercetin 3-rhamnoside
42.11	435.13068 (273)	2.31	$C_{21}H_{24}O_{10}$	Phloridzin
45.58	431.09877	0.92	$C_{21}H_{20}O_{10}$	Kaempferol derivative
47.73	287.05671 (151)	2.08	$C_{15}H_{12}O_6$	Eriodictyol
57.95	273.07751	2.42	$C_{15}H_{14}O_5$	Phloretin

* Identification was confirmed using standard

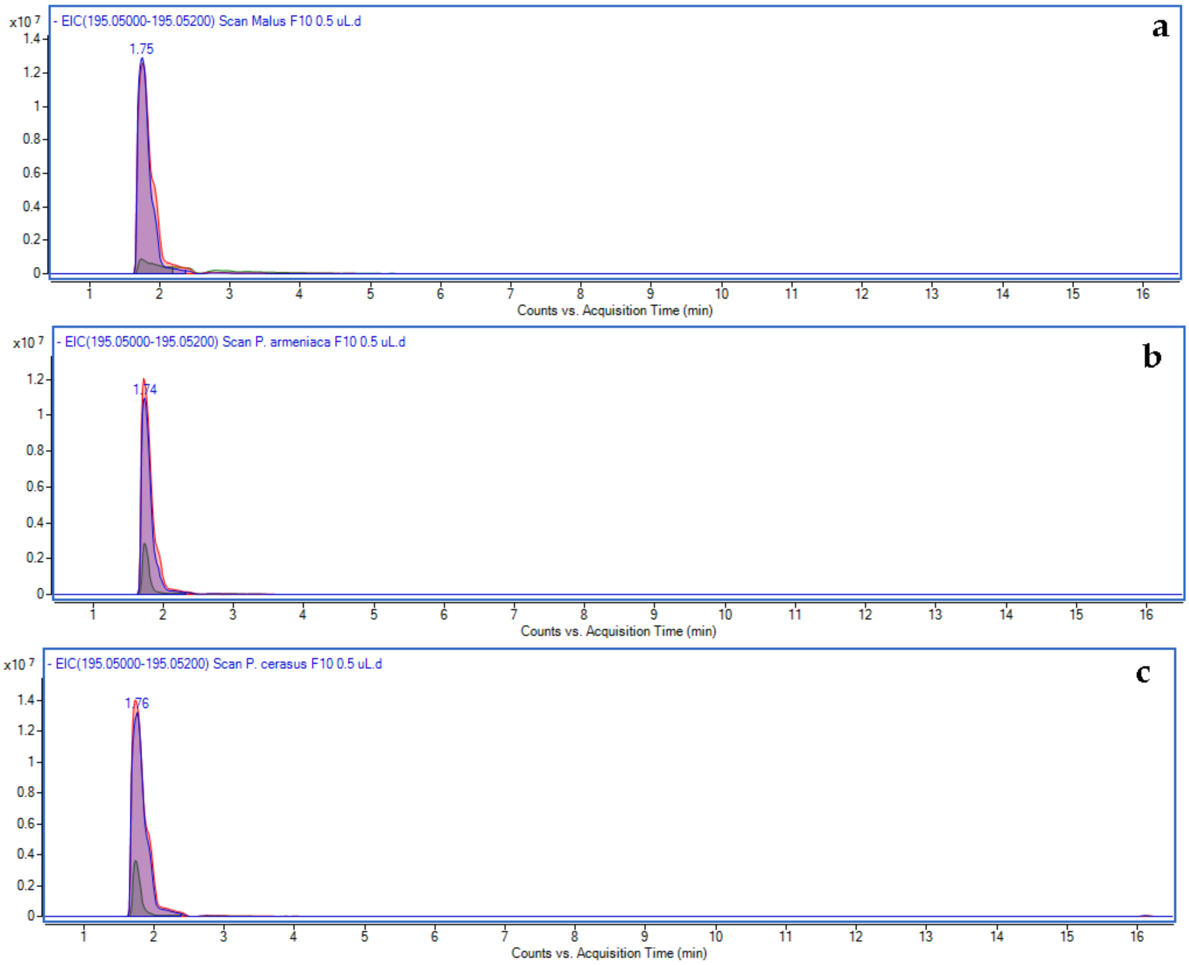

Figure S1. Extracted ion chromatograms (EICs) in the mass range corresponding to gluconic acid from leaf extracts (green line) of (a) *Malus domestica*, (b) *Prunus armeniaca*, and (c) *Prunus cerasus*, overlapped with the corresponding fermented extracts after 10 days of fermentation (blue line) and 20 days (red line).

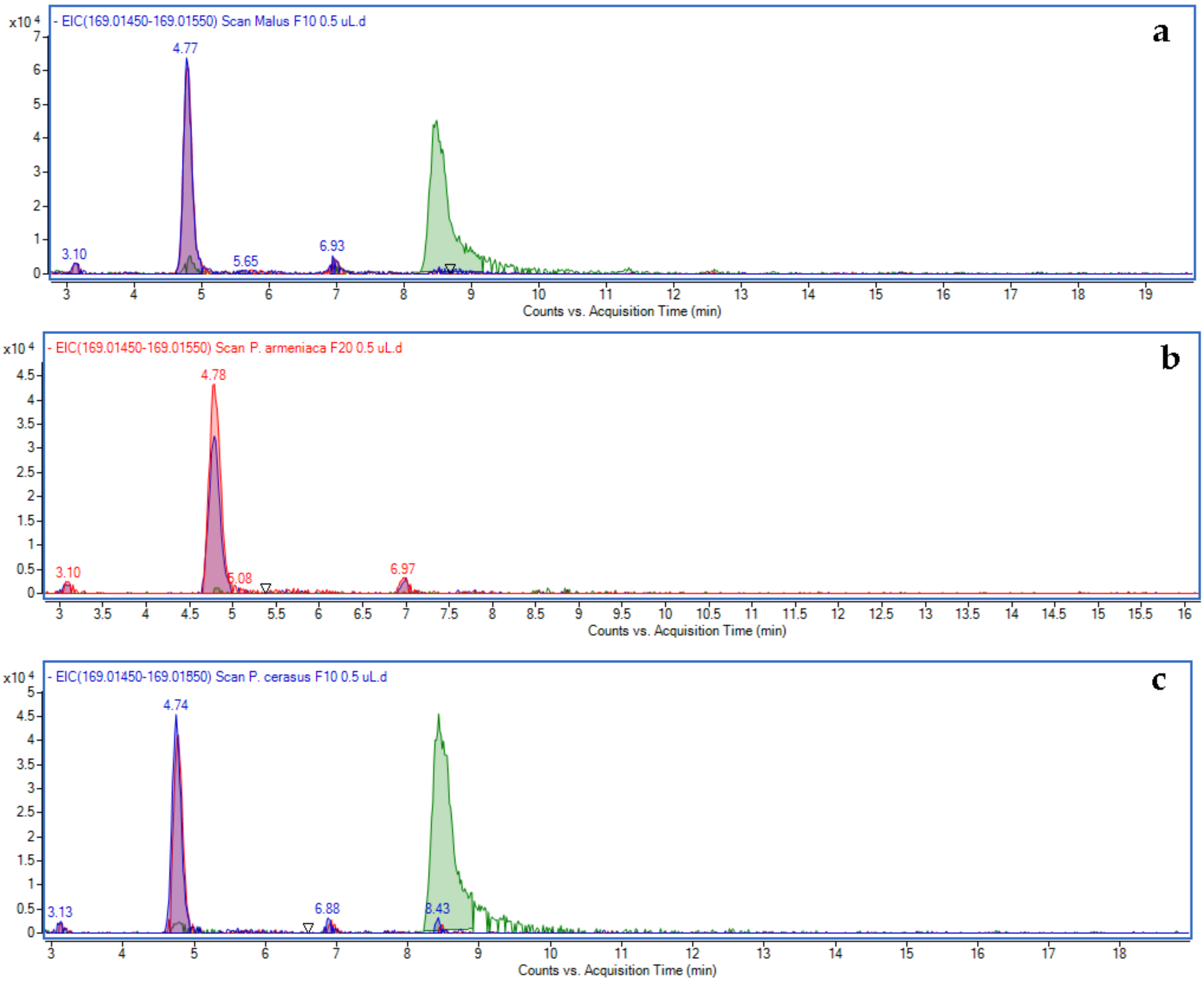

Figure S2. Extracted ion chromatograms (EICs) in the mass range corresponding to gallic acid (retention time=4.7 min) from leaf extracts (green line) of (a) *Malus domestica*, (b) *Prunus armeniaca*, and (c) *Prunus cerasus*, overlapped with the corresponding fermented extracts after 10 days of fermentation (blue line) and 20 days (red line).

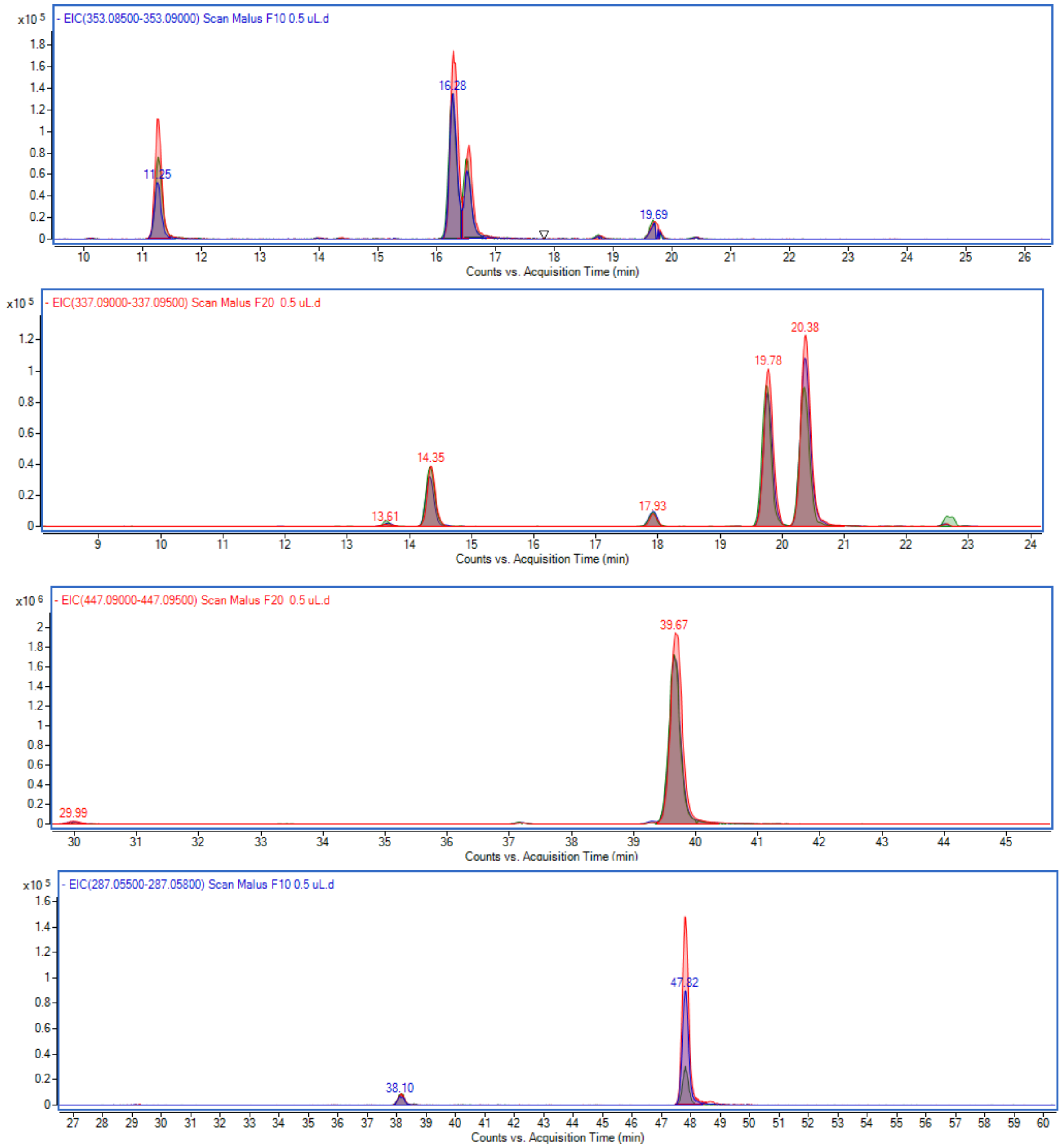

Figure S3. Extracted ion chromatograms (EICs) of components from *Malus domestica* leaf extracts that showed the most significant changes during fermentation: unfermented leaf extract (green line), extract after 10 days of fermentation (blue line), and after 20 days (red line)

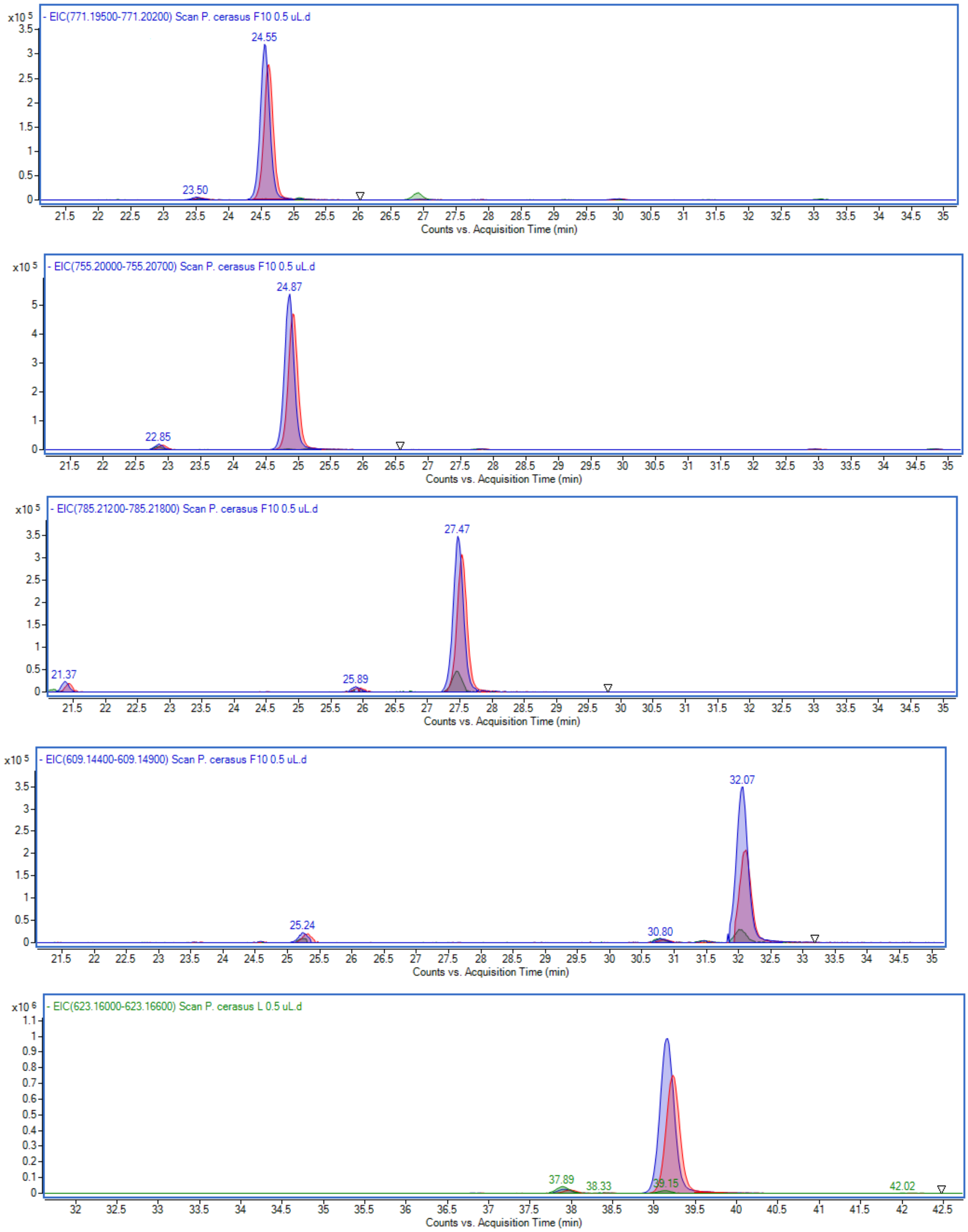

Figure S4. Extracted ion chromatograms (EICs) of components from *Prunus cerasus* leaf extracts that showed the most significant changes during fermentation: unfermented leaf extract (green line), extract after 10 days of fermentation (blue line), and after 20 days (red line).

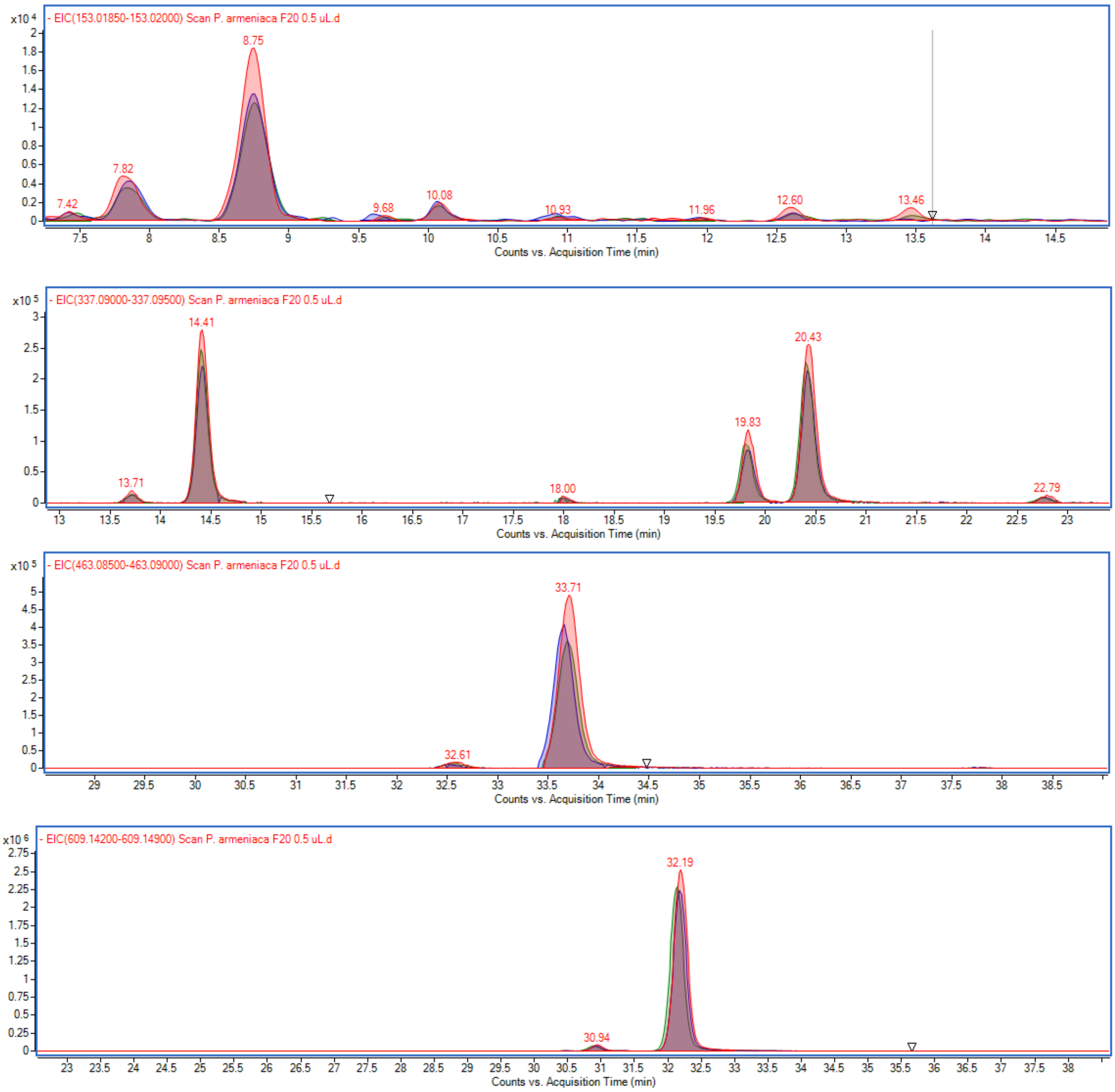

Figure S5. Extracted ion chromatograms (EICs) of components from *Prunus armeniaca* leaf extracts that showed the most significant changes during fermentation: unfermented leaf extract (green line), extract after 10 days of fermentation (blue line), and after 20 days (red line).
